# A Systematic Review of the Effectiveness of Treatments for Depression in Rural and Remote Residents

**DOI:** 10.1002/cpp.70058

**Published:** 2025-03-12

**Authors:** Amelia Bentley, Lucinda Hogan, Jack Howard, Ramnik Singh, Lucinda Watt, Alix Hall, Flora Tzelepis

**Affiliations:** ^1^ School of Medicine and Public Health University of Newcastle Callaghan New South Wales Australia; ^2^ Hunter New England Population Health Hunter New England Local Health District Wallsend New South Wales Australia; ^3^ Hunter Medical Research Institute New Lambton Heights New South Wales Australia

**Keywords:** depression, meta‐analysis, remote, review, rural, treatment

## Abstract

Rural and remote populations have a high burden of depression and poorer access to mental healthcare services than their urban counterparts. This systematic review aimed to assess the effectiveness of psychological and pharmacological treatments on reducing depression specifically in rural and remote residents. Cochrane Library, Medline, PsycInfo, Embase and Scopus, and two clinical trial registries were searched. Included studies were randomised or cluster randomised trials conducted with rural and remote adult populations; examined the effectiveness of any treatment for depression; included a control group or comparator; measured depression; and were published in English. Two authors independently screened records for eligibility, extracted information from eligible studies and assessed risk of bias and certainty of evidence. Seventeen studies were included. Meta‐analyses found a small benefit of behavioural activation therapy (standardised mean difference −0.43, 95% CI −0.78, −0.08, *I* ^2^ = 40%), a large benefit of group therapy (standardised mean difference −1.80, 95% CI −2.80, −0.79, *I* ^2^ = 93%) and no evidence of benefit of interpersonal therapy (standardised mean difference −0.89, 95% CI −2.30, 0.52, *I* ^2^ = 96%) and cognitive behavioural therapy (standardised mean difference −2.39, 95% CI −5.83, 1.05, *I* ^2^ = 98%) for reducing depression in rural populations. Behavioural activation and group therapy appear effective for treating depression among rural populations, although the certainty of evidence is low, and so further research is warranted. Further research on the effectiveness of psychological and pharmacological treatments on depression in rural and remote populations is needed.

**Summary**

- Group therapy appears effective in reducing depression in rural populations.
- Behavioural activation appears effective for treating depression in rural areas.
- Scant evidence for the effectiveness of treating depression with CBT/interpersonal therapy.
- Certainty of evidence was low or very low for depression treatments in rural areas.
- Further research on the effectiveness of treatments for depression in rural areas is needed.

## 1 Introduction

Major depressive disorder (MDD) is defined in the DSM‐5 as ‘episodes of at least 2 weeks duration involving clear‐cut changes in affect, cognition, and neurovegetative functions’ (American Psychiatric Association 2017). A 2019 Global Burden of Disease study revealed that approximately 3.8% of the global population suffers from depressive disorders (GBD 2019 Diseases and Injuries Collaborators 2020).

Studies have examined depression prevalence in rural vs. urban populations, showing mixed findings (Bromet et al. 2011; Peng 2015; Purtle et al. 2019) and increased suicide rates with increased rurality (Lim et al. 2018). Because MDD is a known risk factor for suicide (Caldwell et al. 2004; ‘Mortality Over Regions and Time (MORT) books’ n.d.), higher rural suicide rates may indicate ineffective depression management in these areas.

Access to healthcare involves acceptability, affordability, availability, accessibility and accommodation (McLaughlin and Wyszewianski 2002). Barriers to rural mental healthcare include differences in health resource availability, attitudes toward mental health and rurality's role in seeking help (Caldwell et al. 2004; Kaukiainen and Kõlves 2020; Ferris‐Day et al. 2021). These barriers stem from geographic and social isolation, lack of healthcare options, reliance on external factors and conservative values (Kaukiainen and Kõlves 2020).

The WHO outlines effective depression treatments as psychological and/or pharmacological (WHO 2023). A Cochrane review found sertraline, an SSRI, to be the most effective and well‐tolerated antidepressant (Cipriani et al. 2010). Psychological interventions also show promise in reducing major depression (Nieuwenhuijsen et al. 2020), with varying evidence for the effectiveness of therapies like behavioural therapy, couples therapy, group therapies and interpersonal therapy (Shinohara et al. 2013; Barbato et al. 2018; McDermut et al. 2001; Cuijpers et al. 2011).

Alternative therapies show mixed results: moderate evidence for music therapy, low quality for acupuncture and insufficient evidence for Reiki and dance movement therapies (Aalbers et al. 2017; Smith et al. 2018; Joyce and Herbison 2015; Meekums et al. 2015).

Despite extensive reviews on depression treatment in the general population, evidence for rural and remote populations is limited. A 2015 review found computerised CBT effective for treating depression and/or anxiety in rural residents but called for more research on treatment delivery modalities (Vallury et al. 2015).

Treatments for depression often require GP prescriptions or referrals (Shinohara et al. 2013), and barriers like lack of resources and training for healthcare professionals can hinder treatment in rural areas (WHO 2023). Given the focus on the general population in Cochrane reviews (Barbato et al. 2018; Cipriani et al. 2010; Nieuwenhuijsen et al. 2020; Shinohara et al. 2013; Uphoff et al. 2020) and unique rural barriers, further investigation into effective depression treatments for rural residents is necessary.

This systematic review aimed to assess the effectiveness of psychological and pharmacological treatments on reducing depression in rural and remote residents globally, examining secondary outcomes of adherence to treatment, patient satisfaction and treatment costs.

## 2 Methods

### 2.1 Protocol and Registration

The systematic review protocol was prospectively registered with PROSPERO (CRD42022362270) and reported in accordance with the Preferred Reporting Items for Systematic Reviews and Meta‐Analysis (Page et al. 2021).

### 2.2 Eligibility Criteria

As per the Population, Intervention, Comparison, Outcomes and Study (PICOS) criteria (Amir‐Behghadami and Janati 2020), studies were included if they (i) were conducted with rural and remote adult populations; (ii) examined the effectiveness of any treatment for depression; (iii) included a control group or alternative treatment as a comparator; (iv) measured depression as an outcome; (v) were randomised controlled trials (RCTs), randomised trials, cluster RCTs or cluster randomised trials; and (vi) were published in English. Studies were excluded if they focussed on psychiatric conditions other than depression and/or if the study did not include a specific analysis for the depression outcome for rural/remote participants.

### 2.3 Information Sources and Search

On 28 February 2023, five databases were searched: Cochrane Library, Medline, PsycInfo, Embase and Scopus, as well as clinical trials registries: ANZCTR and ClinicalTrials.gov. Search terms were developed with a University of Newcastle medical librarian and included (i) rural and remote populations, (ii) depression and (iii) randomised study designs. The search strategy also included Medical Subject Headings (MESH) and keywords. The reference lists of included articles were also searched. The search terms used in MEDLINE are displayed in Table 1. Search terms were modified as required for each database.

**TABLE 1:** MEDLINE search terms.

1	Depress*.mp.
2	Depression/or exp Depressive disorder/
3	1 or 2
4	Rural health/ or hospitals, rural/ or rural population/or exp rural health services/
5	Rural.mp.
6	Remote.mp.
7	Regional.mp
8	4 or 5 or 6 or 7
9	Randomi?ed controlled trial.pt.
10	Controlled clinical trial.pt.
11	Randomi?ed.ab.
12	Placebo.ab.
13	Clinical trials as topic.sh.
14	Randomly.ab.
15	Trial.ti.
16	9 or 10 or 11 or 12 or 13 or 14 or 15
17	Exp animals/not humans.sh.
18	16 not 17
19	3 and 8 and 18

### 2.4 Study Selection

The records were imported into Covidence, where all duplicates were automatically removed. Two authors (any combination of AB, JH, LH, LW and RS) performed an independent initial title and abstract screen. Following this, two authors completed independent full‐text screening of all remaining studies and recorded reasoning for exclusion. If a unanimous decision could not be reached during the title and abstract screening, a third author was consulted to review discrepancies. During full‐text screening, all discrepancies were resolved by unanimous decision.

### 2.5 Data Collection Process

A data extraction form was developed based on the Cochrane Effective Practice and Organisation of Care (EPOC) Group's template (Cochrane Effective Practice and Organisation of Care editorial team 2017). Two authors independently extracted the following information from each study: the author(s), year(s) of data collection, country in which the research took place, sample size, participant demographics, recruitment methods, inclusion and exclusion criteria, retention rate, types of interventions and control/comparators, depression scores (primary outcome), adherence to treatment, patient satisfaction with support and costs of treatment (secondary outcomes). Any discrepancies were discussed initially between the two authors. All remaining discrepancies were resolved by unanimous decision.

### 2.6 Risk of Bias (ROB)

Two authors independently assessed the ROB using the Cochrane ROB tool (RoB2) (Higgins et al. 2024). For randomised trials, the RoB2 tool assessed the randomisation process (Domain 1), deviations from intended interventions (Domains 2.1 and 2.2), missing outcome data (Domain 3), measurement of the outcome (Domain 4), selection of the reported result (Domain 5) and overall bias (Domain 6). For cluster trials, an additional domain about the timing of identification or recruitment of participants (Domain 1b) was assessed. Each of these domains was rated as ‘low risk’, ‘high risk’ or ‘some concerns’. Studies rated ‘low risk’ across all domains were classified as ‘low risk’ overall. Studies rated as ‘some concerns’ in one or more domains, but not rated as ‘high risk’ in any domains, were classified as ‘some concerns’ overall. Studies with a ‘high risk’ of bias in any one domain, or “some concerns” in multiple domains, were classified as ‘high risk’ overall. Discrepancies were discussed initially between the two authors, with discrepancies reviewed by a third author.

### 2.7 Synthesis of Results

RevMan 5 was used to conduct meta‐analyses using a random‐effects model, with synthesis stratified by treatment type. For the continuous depression outcome, standardised mean differences (SMDs) were used, given that studies used different measures of depression. SMD values of 0.2–0.49 were considered a small effect, 0.5–0.79 a moderate effect and > 0.8 a large effect (Cohen 2013).

In trials with multiple follow‐ups, the depression outcome recorded at the longest follow‐up assessment was included. *I* ^2^ was used to measure heterogeneity, with *I* ^2^ > 50% representing substantial heterogeneity. For studies that could not be pooled, their effects were summarised alongside the meta‐analysis results. The secondary outcomes were also presented as a summary.

### 2.8 Certainty of Evidence

The Grading of Recommendations, Assessment, Development and Evaluations (GRADE) approach was used to determine the certainty of evidence for the interventions in which a meta‐analysis could be completed. This involved considering the ROB, directness of evidence, heterogeneity, precision and risk of publication bias (Amir‐Behghadami and Janati 2020; Guyatt et al. 2008). Two authors independently used the GRADE framework to rate the certainty of evidence as high, moderate, low or very low. Any discrepancies were discussed initially between the two authors, with discrepancies reviewed by a third.

## 3 Results

### 3.1 Study Selection

**FIGURE 1:**
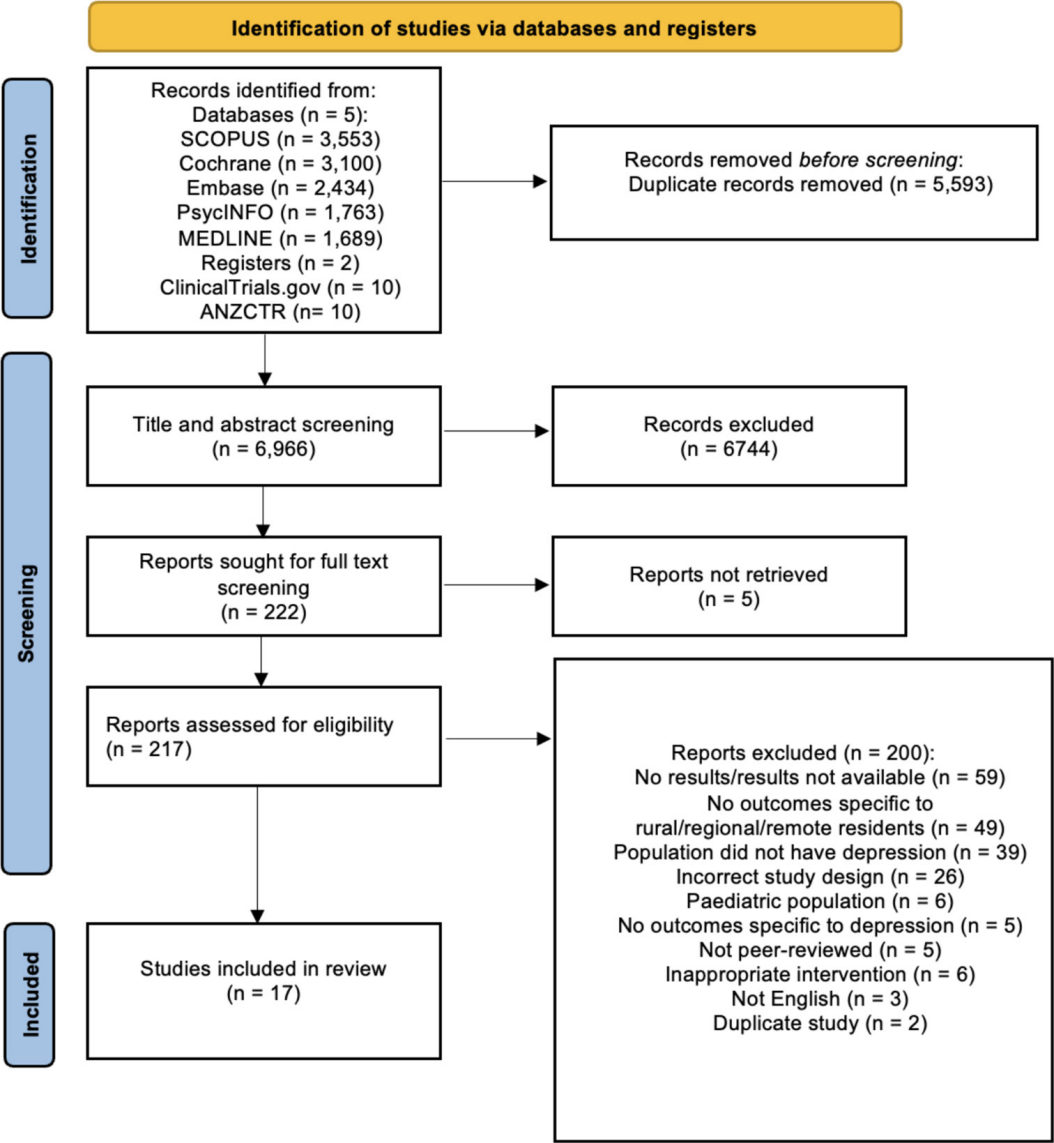
PRISMA flowchart; study selection process.

Figure 1 presents the PRISMA flowchart, which outlines the study selection process. Overall, 12,559 records were identified, and 5593 duplicates were removed, leaving 6966 records for title and abstract screening. A total of 6744 records did not meet the inclusion criteria, and five studies were not located, which left 217 records for full‐text review. During the full text review, 200 texts were excluded for the following reasons: no results/results unavailable (*n* = 59), no outcomes specific to rural/regional/remote residents (*n* = 49), population did not have depression (*n* = 39), incorrect study design (*n* = 26), paediatric population (*n* = 6), no outcomes specific to depression (*n* = 5), not peer‐reviewed (*n* = 5), inappropriate intervention (*n* = 6), not English (*n* = 3), duplicate study (*n* = 2). This left 17 eligible studies.

### 3.2 Study Characteristics

Full details of study characteristics can be found in Table S1.

Of the 17 included studies, the years of publication ranged from 2006 (Bass et al. 2006) to 2022 (Chen et al. 2022). The sample sizes ranged from 40 (Scogin et al. 2018) to 2365 (Chen et al. 2022). Five studies were conducted in the United States (Dwight‐Johnson et al. 2011; Hilty et al. 2007; Ransom et al. 2008; Robinson‐Whelen et al. 2007; Scogin et al. 2018), three in Australia (Almeida et al. 2021; Crockett et al. 2006; Kay‐Lambkin et al. 2012), three in China (Chen et al. 2022; Xie et al. 2019; Yuan et al. 2020) and one study each from Uganda (Bass et al. 2006), Iraq (Bolton et al. 2014), the United Kingdom (Cullum et al. 2007), Bangladesh (Karasz et al. 2021), Nepal (Sangraula et al. 2020) and India (Pradeep et al. 2014). There were 12 RCTs (Almeida et al. 2021; Bolton et al. 2014; Chen et al. 2022; Cullum et al. 2007; Dwight‐Johnson et al. 2011; Hilty et al. 2007; Karasz et al. 2021; Kay‐Lambkin et al. 2012; Ransom et al. 2008; Robinson‐Whelen et al. 2007; Scogin et al. 2018; Xie et al. 2019; Yuan et al. 2020) and 5 cluster RCTs (Bass et al. 2006; Chen et al. 2022; Crockett et al. 2006; Pradeep et al. 2014; Sangraula et al. 2020).

The studies covered diverse interventions. One study (Bolton et al. 2014) looked at a multi‐component intervention; specifically, brief behavioural activation therapy and cognitive processing therapy. Two other studies also examined behavioural activation (Almeida et al. 2021; Xie et al. 2019), four studies CBT (Dwight‐Johnson et al. 2011; Kay‐Lambkin et al. 2012; Scogin et al. 2018; Yuan et al. 2020), four group therapy (Bass et al. 2006; Karasz et al. 2021; Robinson‐Whelen et al. 2007; Sangraula et al. 2020) and two interpersonal therapy (Bass et al. 2006; Ransom et al. 2008). One study each explored disease‐management modules (Hilty et al. 2007), pharmacy‐led interventions (Crockett et al. 2006), liaison‐psychiatric nurse‐led intervention (Cullum et al. 2007), enhanced community care‐worker care (Pradeep et al. 2014) and a collaborative healthcare intervention (Chen et al. 2022).

The tools used to measure depression included Beck Depression Inventory second edition (Hilty et al. 2007; Kay‐Lambkin et al. 2012; Ransom et al. 2008; Robinson‐Whelen et al. 2007), Patient Health Questionnaire 9 (Almeida et al. 2021; Dwight‐Johnson et al. 2011; Karasz et al. 2021; Sangraula et al. 2020), Hopkins Symptoms Checklist (Bass et al. 2006; Bolton et al. 2014; Dwight‐Johnson et al. 2011), Geriatric Depression Scale (Cullum et al. 2007; Xie et al. 2019; Yuan et al. 2020), Kessler Psychological Distress Scale (K10) (Crockett et al. 2006), International Classification of Diseases 10th revision (Cullum et al. 2007), Centre for Epidemiological Studies Depression Scale 10 (Robinson‐Whelen et al. 2007) and Hamilton Depression Rating Scale (HDRS) 35 (Chen et al. 2022; Pradeep et al. 2014; Scogin et al. 2018).

### 3.3 ROB

**FIGURE 2:**
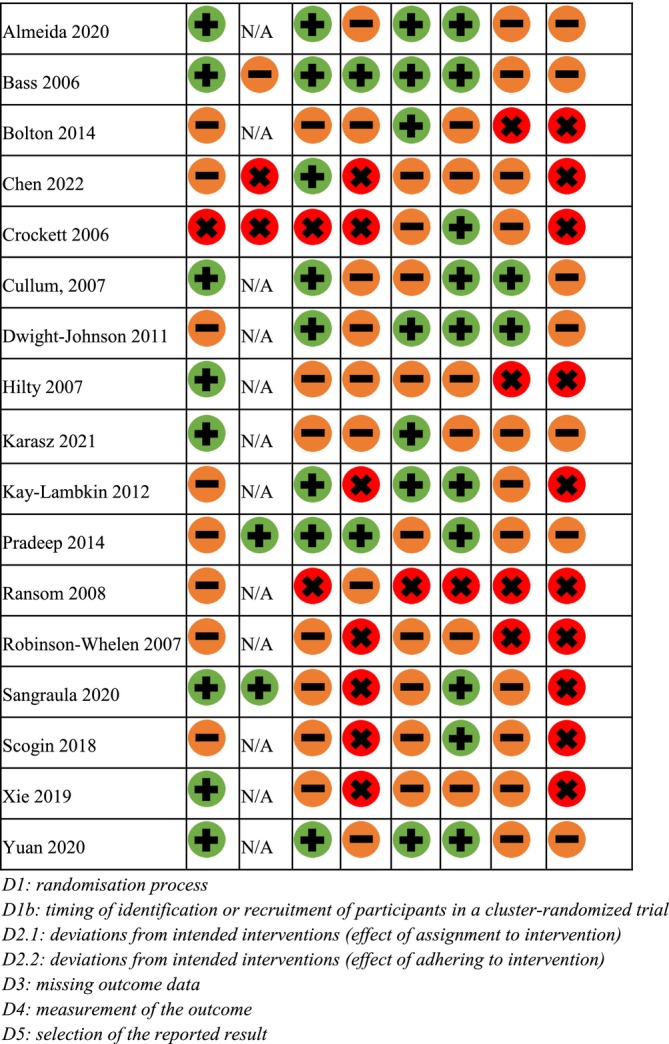
Risk of bias of included studies. D1: randomisation process. D1b: timing of identification or recruitment of participants in a cluster‐randomised trial. D2.1: deviations from intended interventions (effect of assignment to intervention). D2.2: deviations from intended interventions (effect of adhering to intervention). D3: missing outcome data. D4: measurement of the outcome. D5: selection of the reported result.

As shown in Figure 2, the overall ROB was judged as high for 10 studies, whilst seven studies were rated as having some concerns. In Domain 1, eight studies were rated low risk, eight as some concerns and one as high ROB. For cluster randomised trials, in Domain 1b, two studies were rated low risk, one as some concerns and two as high ROB. In Domain 2.1, eight studies were rated low risk, seven as some concerns and two as high ROB. In Domain 2.2, two studies were rated low risk, eight as some concerns and seven as high ROB. In Domain 3, seven studies were rated low risk, nine as some concerns and one as high ROB. In Domain 4, 10 studies were rated low risk, six as some concerns and one as high ROB. Finally, in Domain 5, two studies were rated low risk, 11 as some concerns and four as high ROB. For reasoning of ratings, see Table S2.

### 3.4 Certainty of Evidence (GRADE)

**FIGURE 3:**
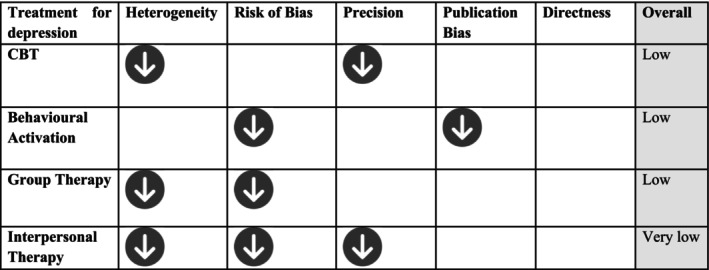
Certainty of evidence for depression treatments.

The certainty of evidence was rated ‘low’ for CBT, behavioural activation and group therapy and as ‘very low’ for interpersonal therapy. Figure 3 illustrates GRADE results and reasons for downgrading the evidence.

### 3.5 Primary Outcomes

#### 3.5.1 Behavioural Activation Therapy

Three studies examined the effectiveness of behavioural activation for reducing depression among rural populations. Almeida et al. (*n* = 308) looked at delivering behavioural activation to elderly rural residents with subthreshold depression symptoms via the post and phone. The control group received booklets at the end of the trial. Both groups were supported by a trained psychologist over the phone. Depression was measured using the PHQ‐9 at 26 and 52 weeks (Almeida et al. 2021).

Xie et al. (*n* = 80) focussed on a rural population with mild to moderate depressive symptoms and compared modified behavioural activation, incorporating elements of cognitive behavioural therapy, to a control group receiving regular care. The Geriatric Depression Scale was used to measure depression at 3 months (Xie et al. 2019).

Bolton et al. (*n* = 281) compared a form of behavioural activation delivered over 12 brief sessions to a waitlist control group. The Hopkins Symptom Checklist (HSCL) measured depression at an average of 5.5 months (range 1.6–15.5 months) (Bolton et al. 2014).

**FIGURE 4:**
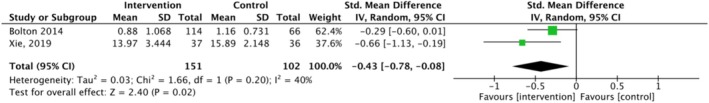
Effectiveness of behavioural activation therapy compared with control in reducing depression.

Two studies (Bolton et al. 2014; Xie et al. 2019) presented the depression outcome in a manner that allowed the results to be pooled in a meta‐analysis (Figure 4). This found a significant difference and small benefit of behavioural activation therapy compared with a control for reducing depression among the rural population (SMD −0.43, 95% CI −0.78, −0.08, two studies, 253 participants, *I* ^2^ = 40% representing acceptable heterogeneity). The certainty of evidence on the effectiveness of behavioural activation therapy on reducing depression was rated low using GRADE (Guyatt et al. 2008).

In the study excluded from the meta‐analysis (Almeida et al. 2021), there was a −2.24 (SD 0.57) point change for the behavioural activation intervention group, compared with a −1.27 (SD 0.56) change for the control.

#### 3.5.2 Interpersonal Therapy

Two RCTs explored the effectiveness of interpersonal therapy on reducing depression among rural residents (Bass et al. 2006; Ransom et al. 2008). One study (Bass et al. 2006) (*n* = 248) allocated participants who met the DSM‐IV criteria for depression to either 16 sessions of group interpersonal therapy or no psychotherapy with access to other supportive care. The HSCL measured depression at 6 months. The other study (Ransom et al. 2008) (*n* = 79) involved participants who resided in areas with less than 50,000 residents, had a self‐reported diagnosis of HIV‐AIDs and met the criteria for a depression spectrum disorder using the Primary Care Evaluation of Mental Disorders (PRIME‐MD). Participants were randomised to either six telephone sessions of interpersonal therapy plus usual care or usual care alone. The Beck Depression Inventory (BDI‐II) was used to measure depression at baseline and after 3 months.

**FIGURE 5:**
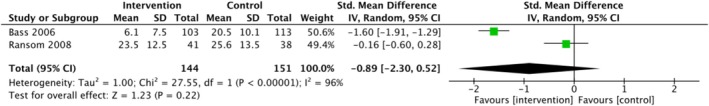
Effectiveness of interpersonal therapy compared with a control in reducing depression.

As shown in Figure 5, a meta‐analysis of the two studies found a non‐significant reduction in depression symptoms for interpersonal therapy compared with a control for reducing depression in rural populations (SMD −0.89, 95% CI −2.30, 0.52, two studies, 295 participants, *I* ^2^ = 96% representing significant heterogeneity). The evidence was rated as very low certainty evidence using GRADE.

#### 3.5.3 CBT

Four studies investigated the effectiveness of CBT on treating depression in rural and remote areas (Dwight‐Johnson et al. 2011; Kay‐Lambkin et al. 2012; Scogin et al. 2018; Yuan et al. 2020). A US study by Dwight‐Johnson et al. with bilingual residents from a rural health setting (*n* = 101) randomised participants to receive either eight CBT sessions via telephone or to a no‐intervention control group. The intervention included behavioural activation. Depression was measured using the PHQ‐9 at 6 weeks and 6 months (Dwight‐Johnson et al. 2011).

Yuan and colleagues conducted a randomised trial in China among rural residents (*n* = 50) who were allocated to either an ‘adapted CBT’ intervention or the usual care control. The adapted CBT intervention addressed somatic complaints with limited use of jargon, and behavioural activations that were relevant to daily life. Depression was assessed with the GDS at baseline, 4 weeks and 8 weeks (Yuan et al. 2020).

In an Australian study, Kay‐Lambkin et al. randomised 53 participants with current comorbid depression, alcohol and/or cannabis misuse into either an intervention delivering computer‐delivered CBT, a clinician‐delivered CBT intervention or the control group, which received person‐centred therapy. Behavioural activation was not involved with either of the interventions. Depression was measured using the BDI‐II at baseline and at 3 months (Kay‐Lambkin et al. 2012).

Scogin et al. randomised 40 participants in the United States to intervention and control groups where the intervention received CBT targeting geriatric depression and insomnia, measured by the HAM‐D at 3 months as a change from baseline (Scogin et al. 2018). The intervention included education on the cognitive‐behaviour mediational model, behavioural activation, and identification and disputing unhelpful thoughts (Scogin et al. 2018).

**FIGURE 6:**
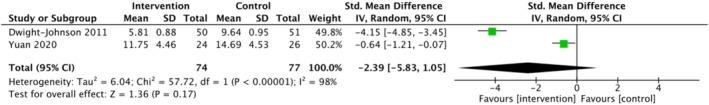
Effectiveness of CBT compared with a control in reducing depression.

Two studies (Dwight‐Johnson et al. 2011; Yuan et al. 2020) were included in a meta‐analysis that found a non‐significant reduction in depression for CBT compared with the control group among the rural population (SMD −2.39, 95% CI −5.83, 1.05, two studies, 151 participants, *I* ^2^ = 98% representing substantial heterogeneity) (Figure 6). The certainty of evidence on the effectiveness of CBT on reducing depression in a rural population was rated as low.

In one study excluded from the meta‐analysis due to insufficient information, at the 3‐month follow‐up, the depression score decreased by 15.52 in the computer‐delivered CBT group, decreased in the clinician‐delivered CBT group by 15.30 and declined in the control group by 10.67 (Kay‐Lambkin et al. 2012).

In the other study excluded from the meta‐analysis due to insufficient detail, the intervention group had a change from baseline on the HAM‐D depression scale of −5.7 at 3 months, whilst the change from baseline for the control group was 2.7 (Scogin et al. 2018).

#### 3.5.4 Group Therapies

Four studies explored the effectiveness of group therapy as a treatment for depression among people living in rural areas (Bass et al. 2006; Karasz et al. 2021; Robinson‐Whelen et al. 2007; Sangraula et al. 2020).

The study by Bass et al. (2006) has been described previously, due to its focus on group interpersonal therapy. Each intervention session involved participants within the group reviewing their current depression symptoms, describing current events in their lives and linking both positive and negative events to their current mood, with support from the other group members. No partners were included.

Karasz et al. (2021) conducted a randomised trial in Bangladesh with rural residents (*n* = 48). Women met fortnightly for 6 months in 12‐person groups, for 12 sessions total, followed by a cash transfer. The intervention included four sessions of financial literacy education and eight sessions of depression treatment, covering mental health literacy, reducing negative cognitions, improving interpersonal relationships and behavioural activation (increasing activity, engaging in pleasurable activities). No partners were included. The PHQ‐9 measured depression at 12 months.

Another study conducted in Nepal (Sangraula et al. 2020) randomised rural residents (*n* = 121) to either five sessions of Group Problem Management (GPM+) or enhanced usual care. Topics covered included managing stress, behavioural activation, managing problems, strengthening social support and reviewing techniques (Dawson et al. 2015). Depression was assessed using the PHQ‐9 at 8–8.5 weeks post‐baseline.

A US study (Robinson‐Whelen et al. 2007) among women with physical disabilities (*n* = 134) compared the effectiveness of an 8‐week depression self‐management therapy group programme led by centres for independent living (CIL) staff members to a usual care control group. The intervention was based on self‐management therapy for depression (Rehm and Adams 2003) and included education on depression, group discussion and weekly homework assignments in which participants monitored their positive activities and daily moods. The intervention involved groups of 10–12 people and incorporated a buddy system. At the 3‐month follow‐up, depression was assessed using the Beck Depression Inventory II (BDI‐II) + 10‐item Centre for Epidemiologic Studies‐Depression Scale (CESD‐10). This study was excluded from the meta‐analysis due to insufficient information. The intervention exhibited improvement of scores at follow‐up. The control group reported higher baseline scores for both measures and also showed an improvement at follow‐up.

**FIGURE 7:**
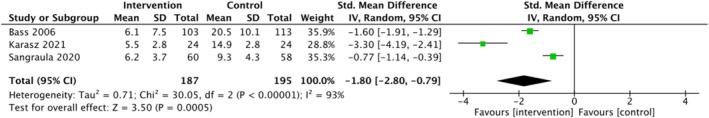
Effectiveness of group therapy compared with a control in reducing depression.

As shown in Figure 7, a meta‐analysis revealed a significant difference and large benefit of group therapy compared with the control group in reducing depression among the rural population (SMD −1.80, 95% CI −2.80, −0.79, three studies, 382 participants, *I* ^2^ = 93% representing substantial heterogeneity). The certainty of the evidence on the effectiveness of group therapy on reducing depression in a rural population was rated as low.

#### 3.5.5 Pharmacist‐Led Treatment

One Australian study (Crockett et al. 2006) compared the effectiveness of a pharmacist‐led intervention to usual care among 119 rural residents. The intervention group was given additional advice and support by pharmacists trained on the nature and management of depression, by a psychiatrist, psychologist and general practitioner, and the control group received usual care. The K10 questionnaire assessed participants' depression. At baseline, mean K10 scores were similar for the intervention (23.0) and control (21.0) groups. There was no statistically significant difference between the K10 scores for the intervention (18.3) and control (17.7) groups at the 2‐month follow‐up.

#### 3.5.6 Liaison‐Psychiatric Nurse‐Led Treatment

One UK study (Cullum et al. 2007) examined the effectiveness of adjunct management by a liaison psychiatric nurse (LPN) who provided social, psychological and medication support. The study randomised rural participants (*n* = 121) to either an intervention group where the LPN formulated a personalised treatment plan for participants or to the usual care control.

At baseline, the mean GDS scores were higher for the intervention group (10.5, 95% CI 10, 11) than the control group (9.6, 95% CI 9.1, 10.1). The mean reduction in GDS at 16 weeks was greater for the intervention group (4.6 reduction) than the control group (3.6 reduction). After adjusting for the difference in baseline scores, these results were not statistically significant.

#### 3.5.7 Community Health Worker (CHW)–Led Treatment

One study conducted in India with rural women (*n* = 280) (Pradeep et al. 2014) examined the effectiveness of enhanced care with CHW intervention for antidepressant adherence and depression severity in comparison to a usual care control. The intervention involved monthly consultations with a primary healthcare physician (PHCP) followed by a CHW home visit where psychoeducation and support were provided to the participant and their family as well as follow‐up of participants who did not adhere to treatment by the CHW to encourage them to continue. The control group was encouraged to seek help from a PHCP and received no additional support. At baseline, mean HDRS scores were similar for the intervention (19.08, SD 4.68) and control (18.99, SD 4.9) groups. At the 6‐month follow‐up, there was no significant difference between the intervention (11.73, SD 7.24) and control (11.30, SD 6.22) groups.

#### 3.5.8 Collaborative Healthcare Approach

One study conducted in China with 2365 rural participants with depression and hypertension (Chen et al. 2022) compared the effectiveness of the collaborative healthcare approach (Chinese Older Adult Collaboration in Health) intervention to enhanced usual care. The intervention featured an algorithm‐driven approach by village primary care doctors supported by village lay workers with telephone consultation from centrally located psychiatrists. Psychiatrists initially visited rural villages to meet intervention group participants in person and then engaged in a monthly review of their case via telehealth with local primary care providers and lay support workers. The control arm received enhanced usual care. Baseline mean HDRS was similar for intervention (22.07, SD 4.52) and control (21.76, SD 3.58) groups. At 12 months, the intervention group (12.69, SD 4.22) had a significantly lower mean HDRS score than the control (18.77, SD 4.67).

#### 3.5.9 Disease Management Modules

One US study (Hilty et al. 2007) randomised participants from rural primary care sites who screened positive for MDD (*n* = 93) to an intervention that implemented intensive disease management modules (IDMMs), which involved education on the biology of depression, regular primary care physician (PCP) visits, telehealth check‐ups from a study nurse and telepsychiatric consultations or to the control group, which received usual care DMM that included visits from the PCP as needed. The Beck Depression Inventory (BDI‐13) and the Symptoms Checklist‐90‐Revised (SCL‐90‐R) assessed depression at baseline and at 3, 6 and 12 months. The results were presented as a graph. Mean scores for both BDI‐13 and SCL‐90‐R at 12 months showed a downtrend in depressive symptom severity for both the intervention and control groups.

### 3.6 Secondary Outcomes

#### 3.6.1 Adherence to Treatment

Only two studies compared the effect of the intervention versus the control group in relation to adherence to treatment (Crockett et al. 2006; Pradeep et al. 2014). The study centred around a pharmacist‐led intervention (Crockett et al. 2006) defined adherence to treatment as the proportion of participants still taking their medication at 2 months, which was 96% for the intervention group and 95% for the control. Furthermore, 20% of participants in the intervention group changed dose or medication during the study compared with 18% in the control group.

Another study examining a community health care worker–led intervention (Pradeep et al. 2014) defined adherence as the average number of visits and average length of treatment. Regarding the number of visits, the intervention group had an average of 3.72 visits (SD 2.35) compared with an average of 1.94 visits (SD 1.24) in the usual care control group. With regards to the length of treatment, the intervention group had an average of 11.1 weeks of treatment (SD 10.4) compared with 3.33 weeks of treatment (SD 3.79) for the control group.

#### 3.6.2 Satisfaction

Two studies compared satisfaction across the conditions (Cullum et al. 2007; Dwight‐Johnson et al. 2011). Cullum and colleagues (Cullum et al. 2007) reported that 93% of participants in a liaison‐psychiatric‐nurse‐led intervention were satisfied with the treatment received compared with 67% in the control group. Dwight‐Johnson et al. (2011) found that 64% of participants offered a CBT intervention reported being very satisfied at 6 months compared with 33% for the control group.

#### 3.6.3 Cost of Treatment

No studies measured the cost of treatment.

## 4 Discussion

### 4.1 Effectiveness of Treatments on Depression

A meta‐analysis revealed a small benefit of behavioural activation therapy compared with the control group in reducing depressive symptoms among a rural population, and the certainty of evidence was rated as low. This is consistent with a systematic review (Uphoff et al. 2020) comparing the effectiveness of behavioural activation therapy to treatment as usual in a general adult population, which showed a similar effect on reducing depressive symptoms.

A meta‐analysis revealed that group therapy had a large benefit in reducing levels of depression when compared with a control group among rural residents. The certainty of evidence was low. This is consistent with evidence from a meta‐analysis (McDermut et al. 2001), which demonstrated an improvement in depressive symptoms among the general population following group therapy. Nonetheless, the results of the group therapy meta‐analysis should be interpreted cautiously due to the small number of included studies. Further research on this topic is needed.

A meta‐analysis found no evidence of the benefit of interpersonal therapy compared with a control for reducing depression in rural and remote communities. However, there was heterogeneity across the populations in the two studies that were pooled in the meta‐analysis on interpersonal therapy, with one study (Bass et al. 2006) including participants who met the DSM‐IV criteria for depression and the other study (Ransom et al. 2008) including participants who had a self‐reported diagnosis of HIV‐AIDS and met the criteria for a depression spectrum disorder using the PRIME‐MD. Given evidence that the prevalence of depression is higher among people diagnosed with HIV compared with the general population (Do et al. 2014), the heterogeneity across the populations in the Bass et al. (2006) and Ransom et al. (2008) studies is a limitation of the meta‐analysis on interpersonal therapy. Furthermore, for interpersonal therapy, the certainty of evidence was rated as very low, which takes into account limitations such as heterogeneity. A meta‐analysis that compared interpersonal therapy with no treatment and usual care among the general population found moderate to large effects of interpersonal therapy for treating depression (Cuijpers et al. 2011). In this meta‐analysis, the overall effect size of the 16 studies comparing interpersonal therapy and a control group was 0.63 (Cuijpers et al. 2011). Given the heterogeneity in the populations across the two studies pooled in our meta‐analysis examining interpersonal therapy and the certainty of evidence rating of very low, there is insufficient evidence to draw reliable conclusions, and further randomised trials are needed to test the effectiveness of interpersonal therapy for depression among rural populations.

Our meta‐analysis revealed no evidence of benefit of CBT compared with a control for reducing depression among rural residents, and the certainty of evidence was low. A systematic review comparing psychological treatments among workers with depression found moderate‐quality evidence that supported the use of CBT as a psychological therapy (Nieuwenhuijsen et al. 2020). Further evidence is required to examine the effectiveness of CBT among rural populations to draw more reliable conclusions about its effectiveness.

Several studies tested other interventions for depression (Chen et al. 2022; Crockett et al. 2006; Cullum et al. 2007; Hilty et al. 2007; Pradeep et al. 2014). Most of these studies did not show a significant benefit in depression outcomes when compared with a usual care control, with the exception of one study (Chen et al. 2022), which suggested that a collaborative healthcare approach may be beneficial in reducing depression in the rural population. Concerns related to the ROB were raised for all of these studies. Additionally, many of these interventions were resource‐intensive and may not be practical for widespread application across geographically diverse locations.

### 4.2 Secondary Outcomes

In relation to the secondary outcomes, only two studies looked at adherence to treatment (Crockett et al. 2006; Pradeep et al. 2014), and two studies examined patient satisfaction with support (Cullum et al. 2007; Dwight‐Johnson et al. 2011). No studies reported on the cost of treatment.

Measurement and reporting of these outcomes were inconsistent between studies, a limitation also faced by an existing review about the feasibility of computerised CBT for depression and/or anxiety in rural areas (Vallury et al. 2015). Vallury et al. (2015) reported that the rurality of residents was unlikely to impact adherence to an intervention, and there were high levels of satisfaction with CBT; however, they discussed diversity between studies. The review did not look at the costs of treatment or other interventions (Vallury et al. 2015).

Given barriers to treatment for depression in rural areas and how these hinder access (Caldwell et al. 2004; Ferris‐Day et al. 2021; Kaukiainen and Kõlves 2020; McLaughlin and Wyszewianski 2002), these secondary outcomes are incredibly relevant when considering treatment approaches in rural settings. Future research needs to allow for a broader perspective when considering study design to focus on effectiveness as well as measuring other features such as adherence, satisfaction and cost, which are also important for decision‐makers to consider.

### 4.3 ROB and Certainty of Evidence

All of the included studies in this review were assessed to have ‘some concerns’ or be of ‘high risk’ of bias. In many of the studies, there was an ROB due to the inability to blind participants to their allocated intervention or control group. Additionally, several studies met the criteria for ‘some concerns’ or ‘high risk’ of bias due to a lack of information regarding the randomisation process, blinding process and data analysis protocol.

The certainty of the evidence for each meta‐analysis was ‘low’ or ‘very low’, highlighting the need for future research, of high quality, to examine the effectiveness of interventions for depression among rural and remote populations.

### 4.4 Strengths and Limitations

Overall limitations for this systematic review were largely related to the availability of studies examining each type of intervention. We were only able to pool a maximum of three studies per meta‐analysis due to the limited evidence examining the effectiveness of each intervention. Moreover, the pooled studies had limited sample sizes, highlighting the need for further studies with larger sample sizes to improve the certainty of the evidence.

Another limitation of the review was that the full text of five studies could not be located during the screening stage and so were excluded. Given our review's focus on rural populations, studies that included urban and rural participants but did not report the outcomes for rural participants only were excluded. A further limitation of the review was that we did not contact authors of these papers to ask if they could provide the findings for the rural subgroup only, which may have eliminated data that otherwise would have been appropriate for this review.

Another limitation of this systematic review, given that we have used global research, was that rurality is a poorly defined concept with multiple definitions in common use. The United Nations broadly separates these definitions into two main categories: ‘area approaches’, where the rurality of a county or municipality is derived from population density and number, and ‘settlement approaches’, where individual settlements are classified as rural or urban based on their size (Goss 2013). Five nations represented in this study use area approaches to define rurality (Australia, ‘Australian Statistical Geography Standard [ASGS] Edition 3’ 2023; Uganda, International Labour Organisation 2003; India, International Labour Organisation 2003; China, International Labour Organisation 2003; and Nepal, International Labour Organisation 2003), whilst the remaining four nations (United States, Health Resources and Services Administration 2022; Bangladesh, Trading Economics 2023; Iraq, Phillips 1959; and the United Kingdom, International Labour Organisation 2003) use a settlement approach. Respective population density, infrastructure and agricultural land‐usage requirements varied between respective countries in these groups. A study examining the willingness of diverse populations to participate in research showed no significant difference between rural/racially diverse and urban populations (McElfish et al. 2018). However, this study reported that the level of education was significantly associated with participation in health research, with those with lower levels of education being less likely to participate in health research (McElfish et al. 2018). Therefore, it is possible that differences in the definitions of rurality across countries may still lead to heterogeneity among the populations (e.g., levels of education) in the studies included in our review and thus may affect the generalisability of the results. The definitions of rurality relevant to our study are included in Table S3.

There is a limitation in the use of eight different depression measures across the included studies. A systematic review (Miller et al. 2021) into the accuracy of depression screening tools found that the PHQ‐9 and BDI‐II (both used in four included studies) met the DSM‐5 criteria for depression. All other measurement tools included in our review did not, with the exception of the Hopkins Symptoms Checklist and the Geriatric Depression Scale, which were not assessed. To account for different depression measures in our review, we used SMDs in the meta‐analyses as recommended in the Cochrane Handbook (Higgins et al. 2024) for studies that assess the same outcome but use different tools to measure depression.

Given that depression measures are often developed in English in western countries, cultural differences and the reliability and validity of such measures with culturally diverse populations must be considered. For instance, cultural variations in depressive symptoms have been found across various countries for Beck Depression Inventory‐II items, including a higher loss of pleasure in Norway, a higher loss of interest in the Dominican Republic and higher sadness in Japan (Seppänen et al. 2022). Although use of the Beck Depression Inventory has been recommended in Europe, there are shortcomings of its use in Spain (Nuevo et al. 2009). Furthermore, there is inconsistent evidence about the cultural appropriateness of the Kessler Psychological Distress Scale (K10) with culturally diverse populations (Stolk et al. 2014). Cross‐cultural adaptation and validation studies have reported that the Geriatric Depression Scale is a valid instrument for assessing depression in Nigeria (Mgbeojedo et al. 2022) and Vietnam (Van Nguyen et al. 2021).

Another limitation of this systematic review was the heterogeneity in the populations across the included studies. Although all studies were conducted with rural and remote adult populations with depression, the participants in some studies also had other conditions such as hypertension (Chen et al. 2022), HIV‐AIDS (Ransom et al. 2008), insomnia (Scogin et al. 2018), alcohol and/or cannabis misuse (Kay‐Lambkin et al. 2012) and physical disabilities (Robinson‐Whelen et al. 2007). The heterogeneity in the populations across studies may influence the findings on the effectiveness of depression treatments in rural and remote populations. The limitation of heterogeneity has been taken into account in the certainty of evidence (GRADE) ratings of low or very low.

Strengths of this study included the use of two independent reviewers during study selection, data extraction, ROB and GRADE assessments and arriving at a majority consensus when the two independent reviewers were unable to reach a unanimous decision. We also completed a very thorough search of literature, including five databases and two registers.

### 4.5 Future Research

There is limited evidence on the effectiveness of all depression treatments in rural and remote areas, and more research is required. Despite the established effectiveness of pharmacological therapy among the general population (Brown et al. 2019), there is no evidence specifically examining the effectiveness of such treatments in rural populations. Future research on the topics explored in our review could particularly focus on additional outcomes important to decision‐making, such as treatment adherence, satisfaction and cost of treatment. There is room for further investigation into the delivery of these therapeutic approaches via virtual methods such as telehealth. The effectiveness of a collaborative healthcare approach to depression management should also be investigated. There is scarce data on the effectiveness of group therapy in rural areas, with the current studies conducted in countries with vastly different social contexts and definitions of rurality (i.e., Nepal, Uganda, and Bangladesh).

Further research could build upon the existing evidence with a focus on improving study designs to minimise the ROB, in particular addressing blinding, attrition rates and following a pre‐established detailed data analysis plan. Additionally, future research conducted with a mix of metropolitan and rural participants could stratify results by urban–rural distribution for the benefit of future reviews focusing on rural populations.

### 4.6 Implications

Our systematic review suggests behavioural activation and group therapy appear beneficial in the management of depression among rural populations. Additionally, group therapy may generally be more cost‐effective than individualised CBT and may be a more appropriate option for depression management in areas with limited resources (Tucker and Oei 2007). Whilst the certainty of evidence was rated as low, group therapy and behavioural activation should be considered by healthcare providers who service rural populations, in conjunction with emerging evidence.

## 5 Conclusion

Meta‐analyses revealed a small benefit for behavioural activation and a large benefit of group therapy on reducing depression among rural populations, although the certainty of evidence is low, and so further research is warranted. Given low and very low certainty evidence for CBT and interpersonal therapy, respectively, further randomised trials examining the effectiveness of these treatments for reducing depression in rural populations are needed. Despite limitations concerning ROB and variability of rurality definitions, treating depression via group therapy and behavioural activation appears to be effective in rural populations. Future research examining the effectiveness of all types of psychological and pharmacological therapies on depression in rural and remote populations is required to strengthen the evidence.

Future research should take into account the barriers to treatment that are unique to rural and remote areas, such as geographic and social isolation, costs of treatment, reliance on external factors, and typically more conservative attitudes and values when examining the effectiveness of treatments for depression in rural populations.

## Ethics Statement

The authors have nothing to report.

## Conflicts of Interest

The authors declare no conflicts of interest.

## Supporting information


**Table S1.** Data extraction table.
**Table S2.** Risk of bias justifications.
**Table S3.** Definitions of rurality.

## Data Availability

Data sharing is not applicable to this article as no new data were created or analysed in this study.
