# Supplementary material for: A Systematic Review of the Effectiveness of Treatments for Depression in Rural and Remote Residents
Source: Clin Psychol Psychother. 2025 Mar 12;32(2):e70058. doi: 10.1002/cpp.70058 (PMC11903104; doi:10.1002/cpp.70058)
Supplement: Supplementary file 1 — Table S1. Data extraction table. Table S2. Risk of bias justifications. Table S3. Definitions of rurality. [file CPP-32-e70058-s001.docx]

| **Supplementary Table 1: Data extraction table** | | | | | | |
| --- | --- | --- | --- | --- | --- | --- |
| **Authors** | **Intervention Type** | **Country**  **Years Data was Collected**  **Sample size**  **Participant demographics** | **Recruitment Method**  **Eligibility Criteria** | **Treatment Arm Descriptions and retention rates for each arm** | **Depression Treatment Outcome measure**  **Follow up timeframe**  **Main results** | **Secondary Outcomes** |
| Almeida et al 2020 | Self-managed behavioural activation therapy | Australia  Time of intervention: not reported  n=307  **Participant demographics**  Intervention:  39% aged 65-69, 33.8% 70-74, 13.6% 75-79, 13.6% 80+  51.3% female, 48.7% male  60.4% married  58.2% previous depressive episode  Control:  46.6% aged 65-69, 27.4% 70-74, 16.3% 75-79, 9.8% 80+  51% female, 49% male  63.2% married  59.5% previous depressive episode | **Recruitment**  Electoral roll was used to identify those over 65 years old, who were then sent a postal pack containing consent, information, and Whooley questions. Those who answered yes to either of the Whooley questions received a phone call to address any doubts about the study, confirm eligibility, and complete assessment for major depressive episode and cognitive status.  **Inclusion criteria**  Aged 65 years or over, screen positive to either of the Whooley questions, reside in regional or remote regions of Western Australia.  **Exclusion criteria**  Active suicidal planning, delusions or hallucinations, sensory impairment that interferes with effective communication over the phone or with reading font size 14, clinically significant cognitive impairment (TICSm < 27), plan to move place of residence during the follow up period, no access to a telephone line (landline or mobile), severe physical illness that compromises 6‐month survival (e.g., terminal cancer) and presence of a major depressive episode at the time of screening. | **Intervention**  Collaborative care, self managed behavioural activation program supported by a trained psychologist who guided the implementation over the phone. Intervention group participants also received a self-help booklet with information, guidance around symptoms, and encouragement to make links between actions and feelings, as well as behavioural management.  113/154 completed trial (20.9% lost to follow up)  **Control**  Participants received the same level of support from study staff, and received self-help booklets at the end of the trial.  121/153 completed trial (26.6% lost to follow up) | **Depression Treatment Outcome measure**  PHQ-9  **Follow up timeframe**  12 months  **Intervention results**  n = 154  mean, (SD)  Baseline: 7.5 (5)  Change in score at follow up: -2.24 (0.57)  **Control results**  n = 153  mean (SD)  Baseline: 7.2 (4.4)  Change in score at follow up: -1.27 (0.56) | Nil relevant |
| Bass et al 2006 | Group Interpersonal Psychotherapy | Uganda  Time of intervention: 2002  n=248  **Participant demographics**  Intervention baseline:  Average age 47.6,  Average 4.7 years of education  50% female, 50% male  85% had major depression  Control baseline: Average age 45.3,  Average 3.9 years of education  53% female, 47% male  94% had major depression | **Recruited method**  Same 30 villages who participated in a 2000 prevalence survey (originally chosen by weighted random sampling based on government census data). For each village a list of eligible people was created and ordered according to decreasing depression severity. Working down the list, eligible individuals were invited to join the study with the goal of recruiting eight participants per village.  **Inclusion Criteria**  Being over 17 years of age, meeting all or all but one of the algorithm’s DSM-IV symptom criteria for depression (subthreshold depression) and reporting some degree of functional impairment.  **Exclusion criteria**  Suicidal ideation, being very different age from rest of group in village. | **Intervention**  Single gender groups of 5-8 participants (one group per village). 90min meetings once weekly for 16 weeks, led by local of same gender, trained in group interpersonal psychotherapy by study team. At each session a group member was invited to review current depression symptoms, describe previous weeks’ events, and link positive and/or negative events to current mood. Group support was encouraged.  Retention at 6 months 103/116 participants or 96%  **Control**  No specific treatment or group meetings. Participants were free to access any locally available treatment or counselling.  Retention at 6 months 113/132 or 97% | **Depression Treatment Outcome measure**  HSCL  **Follow up timeframe**  6 months  **Intervention results** n=103  *mean (SD)*  Baseline: 23.6 (6.5)  6 months: 6.1 (7.5)  **Control results**  n=113  *Mean (SD)*  Baseline: 24.5 (6.1)  6 months: 20.5 (10.1) | Nil relevant |
| Bolton et al 2014 | 1) Brief behavioural activation treatment for depression (BATD)  2) Cognitive processing therapy (CPT) | Iraq  Time of intervention: 2009-2011  n=281  **Participant demographics**  BATD Intervention  Age: mean 36.9 ± 12.4  Sex: 57% female, 43% male  Education (none): 52%  CPT Intervention  Age: mean 41.5 ± 13.7  Sex: 58% female, 42% male  Education (none): 44%  Control  Age: mean 42.3 ± 12.5  Sex: 59% female, 41% male  Education (none): 58% | Participants were identified through referral by doctors and nurses at the participating Ministry of Health primary care clinics and through collaboration with former prisoner organisations who notified their members that the services were available.  **Inclusion criteria:**  Survivors of systematic violence living in the governorates of Erbil or Sulaimaniyah, aged 18 or over, fluent in Sorani Kurdish, reported significant depression symptoms on the adapted HSCL-25, had no current psychotic symptoms or active suicidality, and appeared mentally competent to consent.  **Exclusion criteria**  i) inability to be interviewed due to a cognitive or physical disability  ii) severe suicidal ideation or behaviour | **BATD Intervention:**  A 12-session format which is briefer than other variants of behavioral activation. Core content is based on helping individuals plan for and engage in positive activities on a daily basis based on the values and goals of that individual in multiple life areas. Engagement in these activities is initially supported by the structure of the program and reinforced by the therapist. Two types of adaptations: First, changes based on cultural issues including a) consideration of societal expectations regarding acceptable and unacceptable activities and b) modification of the discussion of values to be less individual-focused and more collective, focused on the participant’s place in the larger society. Second, changes to address patient adversities including limited language/writing proficiency and extreme poverty. These included a set of stickers developed for patients to use in place of written monitoring of activities, and focus on identifying low cost easily accessible activities to reduce financial barriers.  Retention 71.9% (82/114)  **CPT Intervention**  12-session psychotherapy that includes cognitive restructuring and emotional processing of traumatic events. Cognitive restructuring initially focuses on rigid or inaccurate beliefs about the trauma itself. It then shifts to overgeneralized beliefs about the self or other that have been affected by the trauma in the domains of safety, trust, power/control, esteem, and intimacy  Retention 66.3% (67/101)  **Control**  Study participants randomised to the control condition were informed that they would be waitlisted for treatment and then offered the treatment after approximately  5 months regardless of their re-interview results. During the wait period, CMHWs contacted controls monthly to enquire generally about the severity of their symptoms, particularly that they were not a danger to self or others, as a safety check.  Retention 80.3% (53/66) | **Depression and functional impairment measured by adapted Hopkins Symptom Checklist (HSCL)**  with symptom frequency for the prior two-weeks reported using an ordinal scale of 0 (never) to 3 (always). Analyses of intervention impact on mental health outcomes used mean item scores for each scale, therefore also ranging from 0-3.  **Follow-up**  Average 5.5 months (range 1.6 to 15.5 months)  **BATD Intervention (n = 114) - mean (SD)**  Baseline: 1.58 (0.07)  Follow-up: 0.88 (0.10)  **CPT Intervention (n = 101) - mean (SD)**  Baseline: 1.65 (0.07)  Follow-up: 0.89 (0.07)  **Control (n = 66) - mean (SD)**  Baseline: 1.60 (0.04)  Follow-up: 1.16 (0.09) | Nil relevant |
| Chen et al 2022 | Collaborative healthcare approach (**Chinese Older Adult Collaborations in Health (COACH) intervention)** | China  Time of intervention: 2014-2018  n=2365  **Participant demographics**  Mean age: 74.46 (SD 8.23)  Sex: 67% female, 33% male  55% married, 39% widowed  78% lived with a spouse or children  55% had received no education | Recruitment of villages and participants took place over 4 waves. Participants were residents of 218 rural villages located in 10 randomly selected townships of Zhejing Province, China. Each village hosts 1 primary care clinic that serves all residents. Participants were initially identified by review of village clinic electronic medical records by the primary care provider.  **Inclusion criteria:**  i) registration in the village’s primary care clinic  ii) age ≥60 years  iii) a diagnosis of HTN  iv) PHQ-9 score ≥10  v) intact cognitive functioning (Six-Item  Screener [SIS] score <3)  vi) willingness to give written informed consent.  **Exclusion criteria:**  Exclusion criteria included mania, psychosis, or alcohol abuse or dependence active in past 6 months based on the Mini-International Neuropsychiatric Interview (MINI) administered by research assessors at baseline; and acute suicide risk determined by the potential participant’s PCP. | **COACH Intervention -** consisted of algorithm-driven treatment of depression and HTN by village primary care doctors supported by village lay workers with telephone consultation from centrally located psychiatrists. The intervention was administered by a team consisting of the village clinic’s primary care doctor, a village resident called an “Aging Worker” to help reinforce treatment and address social factors affecting health, and telephone-based consultation with a psychiatrist. The psychiatrist provided initial in-person consultation with the PCP in the village clinic and made the initial prescription of antidepressants as indicated. Thereafter, they met with the PCP and AW on a monthly basis by telephone to review cases and make recommendations.  A post-hoc analysis was conducted to assess differences in outcomes for those in the intervention group that refused antidepressant treatment (Antidep -) to those that received antidepressants (Antidep +).  Retention: 93% (1147/1232)  **Enhanced care as usual (eCAU):** CAU is referred to as “enhanced” (eCAU) because PCPs were told when their patients screened positive for depression and were provided with copies of antidepressant treatment guidelines adapted from the Duke Somatic Treatment Algorithm for Geriatric Depression (STAGED)  Retention: 94% (1066/1133) | **Depressive symptoms measured using a validated chinese translation of the Hamilton Depression Rating Scale**  **(HDRS)**  **Follow-up:** 12 months  **Intervention (n = 1232) - mean (SD)**  Baseline: 22.07 (4.52)  Follow-up: 12.69 (4.22)  *> Antidep + (n = 714)*  *Baseline: 22.58 (4.8)*  *Follow-up: 21.76 (4.0)*  *> Antidep - (n = 518)*  *Baseline: 10.7*  *Follow-up: 15.64*  **Control (n = 1133) - mean (SD)**  Baseline: 21.76 (3.58)  Follow-up: 18.77 (4.67) | Nil relevant |
| Crockett Et al2006 | **Pharmacist-led intervention** | Australia  Time of intervention: not reported  n=119  Mean age: 46  **Participant demographics**  Control:  Sex: 82% female, 18% male  Education (with higher than school certificate): 37%  Not currently working (includes pensioners, home duties, unemployed): 50%  Previous depressive episode: 62%  Intervention:  Sex: 76% female, 24% male  Education (with higher than school certificate): 35%  Not currently working (includes pensioners, home duties, unemployed): 63%  Previous depressive episode: 59% | Pharmacists rated between 3 and 6 on the Pharmacy Access and Remoteness Indicator (PhARIA) were asked to watch for antidepressant prescriptions. When a prescription for an antidepressant medication was received, the participating pharmacists were requested to ask the patient what the medication was for, and what the doctor had told them about the medication. If the patient used the word ‘depression’ in their reply and they fitted the selection criteria the patient was eligible for recruitment.  **Inclusion criteria:**  i) receiving antidepressants for "depression"  ii) aged over 18 years  iii) likely to be resident in area for the following three months  iv) patient not aware of a history of psychosis  v) provided consent  **Exclusion criteria:**  i) patient's doctor felt exclusion necessary for medical reasons | **Pharmacist intervention**  Intervention pharmacists were given video-conference training on the nature and management of depression by a psychiatrist, psychologist and general practitioner and asked to dispense medication with extra advice and support.  Retention: 90% (46/51)  **Usual care**  Control pharmacists were asked to provide usual care.  Retention: 88% (60/68) | **Patient psychological wellbeing measured using the K10 questionnaire**  **Follow-up:** 2 months  **Intervention (n = 51, 46 at follow-up) - mean**  Baseline: 23  Follow-up: 18.3  **Control (n = 68, 60 at follow-up) - mean**  Baseline: 21.7  Follow-up: 17.7 | Nil relevant |
| Cullum et al, 2007 | **Liaison psychiatric nurse-led treatment** | United Kingdom  Time of intervention: not reported  n=121  **Participant demographics**  Control:  Mean age (SD): 80.1 (8.07)  Sex: 64% female, 36% male  Marital status (widowed): 59%  Intervention:  Mean age (SD): 79.7 (7.94)  Sex: 53% female, 47% male  Marital status (widowed): 55% | Hospital inpatients were screened for depression rating using GDS-15, ICD-10 depressive disorder derived from the Geriatric Mental State (GMS), previous history of depression (self-rated) and whether or not they were known to the local mental health service identified by search of clinical databases.  **Inclusion criteria:**  i) age ≥65  ii) current residence within the area covered by the PCT and in hospital 3 to 6 days at time of screening  iii) GDS-15 score ≥8  **Exclusion criteria:**  Severe dysphasia, severe deafness, current alcohol dependency or were too physically unwell or confused to participate. Participants who expressed suicidal ideas before randomisation were excluded. | **Liaison psychiatric nurse (LPN) plus usual medical care**. The intervention was implemented by the LPN, who was supervised in the local Community Mental Health Team for Older People (CMHTOP). The LPN assessed patients within 5 days of allocation to the intervention arm and formulated a care/treatment plan. The plan addressed psychological and social needs of the patient, and need for antidepressant medication. The LPN’s role was not to provide all treatments herself, but to liaise with the medical team, primary care, social services and other agencies as well as informal carers to ensure implementation of appropriate management of the patient in hospital and in the community after discharge. The LPN monitored the participant’s mood, mental state and response to treatment every 2–3 weeks for up to 12 weeks, after which the patient was either discharged back to the sole care of their General Practitioner (GP), or to the CMHTOP.  Retention 66.1% (41/62)  **Control - usual care**  Participants in the control arm of the trial received usual care. If the medical team recognised that a patient had depressive disorder possible courses of action would include commencement of antidepressants and/or referral to the mental health service or GP for further assessment and monitoring.  Retention 76.3% (45/59) | **Change in depressive symptoms measured by change in Geriatric Depression Scale (GDS) from baseline + presence of ICD-10 defined depression**  **Follow-up**: 16 weeks  **Intervention (n = 62, 41 at follow-up): - mean (95% CI)**  Baseline: 10.5 (10.0, 11.0)  Mean reduction at follow-up (SD): 4.6 (3.85)  **Control (n = 59, 45 at follow-up): - mean (95% CI)**  Baseline: 9.6 (9.1, 10.1)  Mean reduction at follow-up (SD): 3.6 (3.61) | **Patient satisfaction with care:**  Intervention: 93%  Control: 67% |
| Dwight-Johnson et al 2011 | **CBT via telephone** | USA  Time of intervention: 2011  n=101  **Participant demographics**  Mean age: 40 years  Sex: 78% female, 22% male  92% born in Mexico  77% did not graduate high school  50% employed | Local bilingual residents recruited from the waiting room at a family medical centre in Walla Walla (rural health centre). Clinic providers could also refer.  Inclusion:  Self identified as Latino; spoke English or  Spanish; screened positive for probable major depressive disorder; screened negative for bipolar disorder; minimum of 5-9 symptoms on the PHQ-9, cutoff of 10  Exclusion: Screened positive for bipolar disorder, cognitive impairment, current or lifetime psychotic symptoms or disorder, current substance abuse, or acute suicidal ideation | **Intervention:** Eight telephone sessions of CBT, each focused on a chapter from a patient workbook (translated to Spanish), modified to include latino names and reflect situations relevant to rural Latinos. The workbook included didactic material, exercises for each session, and written exercises for completion between sessions. PHQ-9 assessment at each.  Retention:  **Control:**  Providers were free to provide any usually available care for depression, including antidepressants or referral to outside services. "Enhanced" as participants were encouraged to talk about depression treatment with providers and providers received a letter informing them of their patient's depression status and study enrollment.  Retention: | **Changes in depressive scores as measured by the PHQ-9 and Hopkins symptom checklist (HSCL)**  6 week and 6 month follow ups. 6 month follow ups below.  **Intervention mean scores (SD):**  Baseline: PHQ-9 of 17.02 (0.82) // HSCL of 1.83 (0.12)  Follow up: PHQ-9 of 5.81(0.88) // HSCL of 0.82 (0.12)  **Control mean scores (SD):**  Baseline: PHQ-9 of 17.34 (0.81) // HSCL of 1.75 (0.11)  Follow up: PHQ-9 of 9.64 (0.95) // HSCL of 1.14 (0.13) | **Patient satisfaction (recorded as number of patients who were ‘very satisfied’ at 6 months follow up):**  Intervention N=24/50 (64%)  Control N=12/51 (33%) |
| Hilty et al 2007 | Intensive disease management modules (IDMM) | Hilty et al.  Time of intervention: 2007  USA  n=93  **Participant demographics**  Mean age: 46  Sex: 80% female, 20% male  Married: 44% (control), 54% (intervention)" | Subjects were recruited from rural primary care sites over a 24-month period. PCPs referred subjects with suspected depression for screening. Subjects completed self-report questionnaires and a structured diagnostic interview (i.e., mood and psychotic sections of the Structured Clinical Interview for DSM-IV [SCID-I, research version]) to confirm depression. Subjects with major depression were randomised by the study team.  Inclusion:  "i) English speaking  ii) aged between 18 and 80  iii) willing to take an antidepressant medication  iv) screen positive for major depressive disorder"  Exclusion:  "Subjects without a primary diagnosis of major  Depression based on the semistructured interview was excluded. Subjects with suicidal intention or plans, as demonstrated by a rating of 2 or 3 on the suicide question of the BDI and confirmed by interview, were also excluded and immediately referred to care in their community by communication with the PCP. As has been done in other studies, subjects with dementia, pregnancy, terminal illness, and plans to move in the next 12 months were not enrolled. All other psychiatric and medical disorders were noted for the purposes of analysis, but did not exclude subjects." | Intervention: " Intensive disease management modules (IDMM)  --> usual care with an intensive DMM (IDMM) using telephone, questionnaires, and repeated televideo psychiatric consultation coupled with training of the PCP"  Control: “Usual care disease management modules (DMM)  --> usual care with a DMM using  telephone and self-report  Questionnaires"  Intervention 2: A proportion of the usual care group that received DMM with the initial telepsychiatric evaluation at baseline) | Graph results - probably need to do something further with this but havent at this stage…  For all subjects, baseline BDI-13 scores for the current episode of depression ranged from 8 to 33, with a mean of 18.2 (severe depression). Average scores decreased to 13 (30% reduction), 12 (35%), and 11 (40%) at three, six, and 12 months, respectively (Figure 1). There was no significant difference between the groups, though there was greater variability (i.e., scores wavered much more from baseline) in the DMM group as measured by a permutation test (p<0.05). | Nil relevant |
| Xie et al 2019 | Modified behavioural activation | China  Time of intervention: 2019  n= 80  **Participant demographics**  Mean age: 71.90 ± 3.801,  Sexr: 33 male, 47 female  Schooling: 75% no school completed | **Recruitment**: Recruited by the local health service centre  **Inclusion criteria**  (i) Over 65 years of age (ii) only one participant from each family (iii) left-behind for longer than 6 months (iv) without psychiatric and medical comorbidities that are potentially life threatening or expected to severely limit client participation or adherence (v) are not currently seeing a cognitive–behavioural therapist, psychotherapist or counsellor, including currently receiving antidepressant drug treatment  **Exclusion criteria**  i) GDS score greater than 25 (severe depression) | **Intervention**  Modified behavioural activation treatment:  Intervention used in study was based on behavioural activation (BA) and cognitive behavioural therapy (CBT). The modified model was provided weekly to four groups for intervention, each group including 8–10 participants with 1 facilitator for a period of 8 weeks. A treatment rationale and outline for BA for depression was provided to participants. The daily monitoring form (a blank calendar of the hours of each day of a week) was typically assigned as homework but was started in session to ensure that the participants understood the concepts and  expectations. Strategies such as enlisting a supportive family member to help with written assignments were  encouraged. After submitting the assignment, the local volunteers translated the log and confirmed it with the participants.  Retention rate 92.5% (37/40)  **Control**  Regular care:  Regular care included regular physical  examinations, such as checking the temperature,  blood pressure, heart and breathing rate, and reviewing the current health symptoms depending on their health needs, and receiving same education contents  (knowledge on the preventions and treatments of chronic diseases, infection diseases, etc.) delivered by village doctors weekly during the 8-week intervention period.  Retention rate 90% (36/40) | **Change in severity of depression symptoms measured by Geriatric Depression Scale (GDS) at baseline and 3 months.**  **Intervention**  Baseline: 16.14 (1.888)  3 months: 13.97  **Control**  Baseline 15.78 (1.476)  3 months: 15.89 (2.148) | Nil relevant |
| Karasz et al. 2021 | Group therapy – Fortnightly depression management and  financial literacy intervention | Bangladesh  Time of intervention: 2021  n=48  **Participant demographics**  Intervention  Means age: 26  Mean education: 6 years education at school  4.63 people per family on average.  Control  Mean age: 26.1  Mean education: 4.4 years education at school  Mean 5.2 people per family | Over a two-month period, peer workers visited homes in the two villages and screened  163 housewives  Inclusion criteria included the following:  housewife;  age between 18 and 40 years;  depressive symptoms (Patient Health Questionnaire (PHQ-9) score ≥ 10);  minimum literacy (participants were asked to read a short paragraph and write a sentence);  ability to save a minimum of 50 takas (around $0.60/week).  Exclusion:  Very low literacy and numeracy,  enrolled in other studies,  husband refused permission,  have debts and cannot save | Intervention: The intervention included a six-month group-based, fortnightly depression management and  financial literacy intervention, which was followed by a cash-transfer of $186 (equivalent to the cost  of two goats) at 12 months’ follow-up. The cash transfer could be used to purchase a productive asset  (e.g., agricultural animals)  Control: the control group received no treatment  Only participants who completed the follow up were recorded in baseline data | **Changes in depressive symptoms measured by PHQ-9**  12 month follow up  **Intervention**  Baseline: 14.5  Follow up: 5.5  **Control**  Baseline: 14.5  Follow up: 14.9 | Nil relevant |
| Kay-Lambkin et al. 2012 | Therapist-delivered cognitive behavioral therapy/motivational interviewing | Australia  Time of intervention: 2012  n=53  **Participant demographics**  Rural participants  Mean age: 36.65  Sex: 55% females, 45% males,  Employment: 51% employed | Recruited from The SHADE study which gathered participants with current comorbid depression and alcohol and/or cannabis misuse. The sample was drawn from rural and urban locations across New South Wales, Australia.  Inclusion:  Rural or urban NSW dwelling currently experiencing moderate levels of depression (17 or higher on the Beck Depression Inventory  II) and were concurrently using alcohol above Australian national guidelines (greater than 40 g daily ethanol for men or 20 g for women) and/or cannabis more than once weekly for the month prior to baseline as measured by the Opiate Treatment Index.  Participants were eligible for the current analysis if they completed the 3-month post-baseline assessment  N = 53 for follow up | 163/274 participants did the 3 month follow up - only these participant’s data was reported  Intervention 1:  All three treatments were 10 sessions, completed over  10 to 15 weeks, with session 1 identical across conditions and  delivered face-to-face. **+ 9-session therapist-delivered CBT = ; Therapist CBT/MI = therapist-delivered cognitive behavioral therapy/motivational interviewing**  **Intervention 2:**  All three treatments were 10 sessions, completed over  10 to 15 weeks, with session 1 identical across conditions and  delivered face-to-face. **+ 9-session computer-delivered CBT = CAC CBT/MI = clinician-assisted**  **computerised cognitive behavioral therapy/motivational interviewing**  **Control:**  **9-session Person Centred Therapy = SC = control.** | **Changes in depression severity measured by the BDI-II**  **Intervention one - therapist CBT/MI**  Baseline: 33.02  Follow up: -15.30 change  **Intervention two - CAC CBT/MI**  Baseline: 33.02  Follow up: -15.52 change  **Control**  Baseline: 33.02  Follow up: -10.67 change | Nil relevant |
| Pradeep et al. 2014 | Intervention with community health workers to improved medication adherence | India  Time of intervention: 2006 - 2009  n=280  **Participant demographics**  Marital status and family: 87.3% married, 57.3% from nuclear families  Education and employment: 56% were not formally educated and 94.5% were not employed in formal/informal sector | 814 houses were surveyed in six villages with a trained research investigator screening rural women for depression  Inclusion criteria: Rural women, treatment naive depression diagnosis, informed consent NB - depression diagnosis GHQ and then mini international neuropsychiatric interview for diagnosis  Exclusion criteria: treatment last 6 months | Intervention: Patients diagnosed  with depression needed to visit the primary health  centre once a month to consult with the physician. Community health workers visited patients immediately following the first medical consultation, educated the patient and her family members about depression and its treatment. They also emphasised taking antidepressant medication and continuing the treatment regimen. This was followed by another visit in the subsequent week to enquire about any possible side effects of medication and clarification of any doubts concerning the medical treatment of depression. This pattern of visits was maintained after every monthly consultation with the physician in the intervention group. In addition, community health workers visited those patients who discontinued medication  and / or those who did not visit the PHC for an initial  consultation and encouraged them to resume treatment in the intervention group.  → retention rate 100%  Control: treatment as usual – patients diagnosed with depression were encouraged to seek help from the physician at PHC with no additional input from the CHW.  → retention rate 100% | **Symptom severity measured using the Hamilton Depression Rating Scale (HDRS)**  6 month follow up  **Intervention group:**  Baseline: 19.08  6 month F/U: 11.73  **Control group:**  Baseline: 18.99  6 month F/U: 11.30 | Adherence is defined as the average number of visits and average length of treatment.  **Number of visits:**  **Intervention group:**  3.72 (2.35)  **Treatment as usual group:**  1.94 (1.24)  **Length of treatment (mean, SD)**  **Intervention group:(mean, SD)**  11.1 (10.4)  **Treatment as usual group:**  Baseline:  3.33 (3.79) |
| Ransom et al. 2008 | Interpersonal therapy – Telephone psychotherapy | USA  Time of intervention: 2008  n=79  **Participant demographics**  Mean age (SD): 44.4 ± 8.6 years  Sex: 16% female, 84% male  Sixty-one were white (77%), eight were African American (nonHispanic) (10%), seven were Latino (9%), two were African American (Hispanic) (3%), and one was Native American (1%).  The average participant had completed 12.2±1.9 years of formal education  At pre intervention, participants’ mean BDI-II score was 27.4±11.0, with 68 (86%) reporting BDI-II scores higher than 13  February 2006 to March 2007 | Seventy-nine participants were recruited through AIDS service organisations in ten states. Recruitment packets containing information about the study, eligibility criteria, and toll free contact information were mailed to 21 AIDS service organisations; the agencies then forwarded packets to potential participants.  Inclusion:  1) 18 years of age or older 2) self-reported diagnosis of HIV-AIDS 3) voluntary provision of informed consent 4) residence in a community with 50,000 residents or fewer (that is, the criteria used by the Centers for Disease Control and Prevention to designate communities as nonmetropolitan 5) met diagnostic criteria for a depression-spectrum disorder, as measured by a telephone-administered version of the Mood Module of the Primary Care Evaluation of Mental Disorders (PRIME-MD) - specifically, participants met criteria for a major depressive episode or dysthymic disorder | Intervention: Interpersonal therapy intervention plus usual care. Participants assigned to the teletherapy condition (N=41) received six 50-minute sessions of telephone-delivered therapy. Similar to participants in the usual care group, those in the teletherapy group had access to services provided by their AIDS service organisation and other providers in the community. At the start of the intervention, all participants in the teletherapy group were encouraged to identify a private location for sessions, schedule sessions at a time when few interruptions were anticipated, and refrain from the use of call waiting, except in possible emergency circumstances.  → retention: 31/41 = 76%  Control: Received no active therapeutic intervention.  → retention: 35/38 = 92% | **Changes in depression scores measured by the BDI-II**  Follow up post study only  **Intervention**  Baseline: 28.7  Follow up: 23.5  **Control:**  Baseline: 26.1  Follow up: 25.6 | Nil relevant |
| Robinson-Whelen et al. 2013 | Group therapy – 8-week depression self-management therapy program | USA  Time of intervention: 2007  **Participant demographics**  n=134  mean age: 51.59, 79.2% non-hispanic white, 39.6% married, 25% employed, disability duration mean 15.09 years, mean age at onset of disability 36.5 | Women with physical disabilities were recruited through centres for independent living (CILs) to participate in the depression self-management study. included announcements in print and radio media as well as notification about the study to the CIL consumers, churches, doctors' offices, and other agencies. These announcements stated that the study was designed to address depression among rural women with physical disabilities.  Inclusion:  (a) were at least 18 years of age (b) had a health condition causing a significant limitation in mobility or self-care (c) had had their disabilities for at least 1 year (d) scored 14 or higher on the BDI–II at the screening interview  Exclusion:  (a) presented with active suicidality (b) presented with or reported recent psychotic symptoms (e.g., hallucinations) that could interfere with participation (c) did not meet a cut-off score of 12 or greater on a brief cognitive screen consisting of items (i.e., orientation to time, 5 points; orientation to place, 5 points; registration of three words, 3 points; and recall of three words, 3 points) from the Mini-Mental State Examination (d) reported a suicide attempt in the previous 10 years and had a score of 30 or higher on the BDI–II | Intervention: An 8-week depression self-management therapy program led by CIL staff members who received pre- intervention training and ongoing clinical supervision.  → retention: 42/69 (60%) completed at least 4/8 intervention group sessions + the follow up  Control: usual care  → retention: 54/65 (83%) completed initial test and the follow up | **Changes in scores of Beck Depression Inventory II (BDI-II) + 10-item Centre for Epidemiologic Studies-Depression Scale (CESD-10)**  3 month follow up  **Intervention**  Baseline: BDI-II of 25.20 // CESD-10 of 16.61  Follow up: BDI-II of 17.58 // CESD-10 of 14.77  **Control**  Baseline: BDI-II of 29.07 // CESD-10 of 19.10  Follow up: BDI-II of 24.88 // CESD-10 of 17.18 | Nil relevant |
| Scogin et al. 2018 | Integrated CBT | USA  Time of intervention: 2018  Unknown  n=40  **Participant demographics**  Age: mean 58.32 ± 6.69 (intervention), 59.78 ± 8.50 (control),  Gender: Female:male 18:4 (intervention), 18:0 (control) | Recruitment  Physician and nurse referrals, medical record surveys and brief waiting room questionnaires  Inclusion criteria  (1) being 50 years of age or older (2) a resident of Alabama’s Black Belt and receiving services from one of five primary care collaborators (3) absence of significant cognitive impairment as indicated by a score of 20 or higher on the Saint Louis University Mental Status Examination (4) absence of other sleep disorders (5) not currently receiving psychological treatment (6) absence of suicidality (7) absence of a self-reported psychotic disorder or substance dependence or abuse (8) concurrence from patient’s primary care physician indicating presence of both insomnia and depression symptoms of sufficient significance to warrant initiation or continuance of primary care treatment | Intervention  A refined 10-session manualised protocol based on evidence-based treatments for geriatric depression and geriatric insomnia. The three depression components were: 1) presentation of the cognitive behaviour mediational model, 2) behavioural activation 3) identification and disputation of unhelpful thoughts. Roughly 25 minutes of each treatment session was devoted to CBT for depression and 25 minutes to CBT for insomnia.  27% retention rate (6/22 provided results for final follow up)  Control  Physician-recommended primary care services for insomnia/depression. This may include pharmacotherapy for sleep and depression as well as psychiatric referral.  44% retention rate (8/18 provided results for final follow up) | **Change in severity of depressive symptoms measured by the Hamilton Depression Rating Scale (HAM-D) at baseline, completion of treatment/control and at 3 month follow up.**  No raw data on baseline and follow up scores for intervention or control.  **Intervention:**  Change from baseline: -5.7 (SD = 1.9)  **Control:**  Change from baseline: 2.7 (SD = 2.0) | Nil relevant |
| Suarangurala et al. 2020 | Group therapy – Problem management plus | Nepal  Time of intervention: 2020  n=121 (61 intervention, 60 control)  **Participant demographics**  Age 46.7 ± 14.0 (intervention), 49.2 ± 13.6 (enhanced usual care)  Sex:  83% female, 17% male (intervention) 84% female, 16% male (enhanced usual care) | No data on recruitment  **Inclusion criteria**  1) 18 years of age and older 2) score of >2 on the General Health Questionnaire (GHQ) 3) score of >16 on the WHO Disability Assessment Schedule 2.0 (WHODAS)  **Exclusion criteria**  1) symptoms of psychosis and severe cognitive impairment 2) AUDIT score >16 | **Intervention**  Problem management plus (PM+):  Five sessions of Group PM+, each session lasting 2.5–3 hours. Sessions included: (1) Managing Stress, (2) Behavioural Activation, (3) Managing Problems, (4) Strengthening Social Support and (5) Review of Technique. There were ten groups in the Group PM+ arm. Participants were allocated to groups based on their location.  The groups consisted of six to eight people separated by gender and with gender matched facilitators. Local volunteers supported facilitators by organising logistics and reminding participants about the sessions.Community based psychosocial workers (CPSW) were the service providers for the groups  Retention rate 60/61  **Control**  Enhanced usual care: CPSWs delivered family meetings to participants in both arms. This consisted of: (a) consent to take part in the study and follow up assessments, (b) psychoeducation on adversity, (c) benefits from support, (d) information on the availability of mental health services by a mental health Gap Action Programme Intervention Guide (mhGAP)-trained health worker in the nearby clinic.  Retention rate 58/60 | **Change in severity of depression symptoms measured by Patient Health Questionnaire (PHQ-9) at 8-8.5 weeks post baseline.**  **Intervention**  Mean at baseline: 9.8 (4.9)  Mean at follow up: 6.2 (3.7)  **Control**  Mean at baseline: 10.7 (4.4)  Mean at follow up: 9.3 (4.3)  This data includes patients lost to follow up | Nil relevant |
| Yuan et al. 2020 | Adapted CBT | China  Time of intervention 2020  n=50  **Participant demographics**  Age: mean 70.5 ± 5.6 years, range 64–90  Sex: 24 female, 26 male | **Recruitment**  No information  **Inclusion criteria**  (a) 60 years old or older;  (b) CES-D score >15;  (c) GDS score >9; and  (d) MMSE score >17  **Exclusion criteria**  (a) lifetime  psychosis;  (b) significant suicide risk; and  (c) poor physical condition (e.g. cancer, dementia)  or impairment in communication | **Intervention**  Adapted CBT: Adaptations include (1) counsellors were suggested or encouraged to flexibly replace jargon in CBT with dialectics, proverbs and other attuned words (2) older adults decided the place (home/ clinic); (3) each session started with a physical health examination to address somatic complaints; (4) behavioural activations were tailored to improve adherence in daily life Health workers (monitored) delivered the eight, 45-minute, weekly individual therapies. A licensed psychologist held a weekly, face-to-face group supervision and provided individual urgent supervision.  Retention rate 79% (19/24)  **Control**  Usual care: Usual care was door-to-door chronic disease management for all older adults or out-patient services in clinics. Counsellors were encouraged to provide psychosocial education and referrals for CAU participants with severe mental problems.  Retention 100% (26/26) | **Geriatric depression scale measured at baseline, 4 weeks, and 8 weeks.**  **Intervention mean**  Baseline mean 13.79 (SD 3.46)  Follow up mean 11.75 (SD 4.46)  **Control**  Baseline mean 14.65 (SD 3.42)  Follow up mean 14.69 (SD 4.53) | Nil relevant |
| Abbreviations:PHQ-9 - Patient Health Questionnaire 9HSCL - Hopkins Symptom ChecklistK10 - Kessler Psychological Distress Scale | | | | | | |

##

| **Supplementary Table 2 - Risk of bias justifications** | | | | | | | | |
| --- | --- | --- | --- | --- | --- | --- | --- | --- |
|  | **D1** | **D1b (cluster RCTs)** | **D2.1** | **D2.2** | **D3** | **D4** | **D5** | **Overall** |
| **Almeida et al. 2020** | **Low risk**    Randomisation completed by random sequence. Blinded until after allocation. Characteristics of participants balanced across groups | **NA** | **Low risk**  Participants could not be blinded to intervention, however there is no evidence this affected results. Analysed using intention to treat. | **Some concerns**  Participants and implementers of programs were aware of assigned intervention. No information given on adherence to interventions, but intention to treat analysis used. | **Low risk**  High dropout rates in both intervention arms, however, used intention to treat analysis. | **Low risk**  Evidence based scoring system used for results, which were ascertained identically from each group by a implementer blind to intervention arms | **Some concerns**  All eligible analyses of the data included, however no locatable study protocol or analysis plan | **Some concerns**  Concerns raised in domains D2.2 and D5. As these are both raised concerns due to lack of information rather than inappropriate analysis/methods - not high risk. |
| **Bass et al. 2006** | **Low risk**  Randomisation of villages previously chosen by random sampling. Allocation concealed until allocation. No evidence of bias in intervention demographics | **Some concerns**  Randomisation by village, but no information if this was done before or after recruitment of participants. Nil baseline imbalances to suggest selection of participants was affected | **Low risk**  Participants could not be blinded to intervention, however there is no evidence this affected results. Intention to treat analysis used. | **Low risk**  Participants were aware of assigned intervention, however adherence to interventions was comparable. | **Low risk**  Data available for nearly all participants included. Dropout rates are low and equal across groups. | **Low risk**  All interviews happened in the same location, with interviewers blinded to interventions received, and all received the same assessments. | **Some concerns**  All eligible analyses of the data included, however no locatable study protocol or analysis plan | **Some concerns**  Some concerns due to the fact that domain D1b and D5 have raised some concerns. |
| **Bolton et al. 2014** | **Some concerns**  Double randomisation, workers were randomised before participants and were aware of their intervention before randomisation was complete. No information on if allocation was concealed but no significant baseline imbalances | **NA** | **Some concerns**  Change in study design with deviations in control groups + possibility of contamination of data. These deviations are probably balanced across both control groups.  Post-hoc analysis was carried out to assess the impact of this. | **Some concerns**  Change in study design with deviations in control groups + possibility of contamination of data. Personnel aware of intervention. Appropriate analysis was carried out post-hoc to analyse the effect of this. | **Low risk**  Intention to treat analysis with sensitivity analysis conducted to assess for bias in results | **Some concerns**  15% of follow-up assessments were conducted by non-blinded outcome assessors. It is considered possible but unlikely that assessment was influenced by knowledge of the intervention. | **High risk**  High risk of bias due to multiple analyses being conducted on the same data (ie the post-hoc adjustment due to the unexpected heterogeneity of the cohort) | **High risk**  High risk overall as domain 5 is high risk. There are significant concerns regarding the potential contamination of results between clusters, with the authors of the study conducting a second post-hoc analysis to attempt to correct for this. |
| **Chen et al. 2022** | **Some concerns**  Groups had several statistically significant differences at baseline (namely in psychological wellbeing, quality of life and employment status). These suggest that there probably was a problem at some point in the randomisation process. | **High risk**  Randomisation occurred prior to recruitment of participants. Recruiters were aware of allocation to intervention or control. There is a likelihood that this may have subconsciously affected assessment of participants for eligibility in the trial. | **Low risk**  While masking was not possible for assessors and participants, all were blinded to the study objectives and hypothesis. No deviations because of the trial context are evident and appropriate analysis was conducted. | **High risk**  Significant non-adherence in the intervention group that was not adequately accounted for in analysis to determine the effect on the outcome. | **Some concerns**  It is possible (but not likely) that some missingness of the data could be related to depressive symptoms (i.e. dependent on the true value). | **Some concerns**  Researchers were aware of the allocation for villages they were assessing, however it is unlikely (but still possible) that this would have affected results. | **Some concerns**  No information provided in the study protocol for a pre-specified analysis plan. | **High risk**  High risk overall as domain D1b and 2.2 is high risk. The adherence rates of the intervention group were ambiguously reported, but significant. While subgroup analysis was conducted on the proportion that did not take antidepressants, there was no adequate statistical analysis (e.g. sensitivity analyses) conducted to account for potential bias. |
| **Crockett et al. 2006** | **High risk**  Pharmacists were randomised to intervention or control before they recruited participants (ie allocation sequence not concealed) | **High risk**  Randomisation occurred prior to recruitment of participants. Recruiters were aware of allocation to intervention or control. There is a likelihood that this may have subconsciously affected assessment of participants for eligibility in the trial. | **High risk**  4 of the pharmacists assigned to the control arm were found to be delivering a service to their patients which paralleled that being provided by the  intervention pharmacists. This had the potential to confound the results. | **High risk**  4 pharmacists assigned to the control arm provided services equivalent to the intervention arm. No evidence of appropriate analysis to determine the effect of adhering. | **Some concerns**  Outcome data not available for all participants, with no evidence that the result is not biassed as a result. It is considered unlikely that missingness was dependent on the true value. | **Low risk**  No information if a mental health nurse assessor was blinded to participant allocation, however it is considered unlikely that knowledge of allocation could have affected the result of a self-report questionnaire. | **Some concerns**  No information provided regarding a pre-specified data analysis plan. Study protocol unavailable for reference. | **High risk**  High risk overall as domains , 1b, 2.1 and 2.2 have a high risk for bias. Concerns are raised over the potential for bias in the recruitment stages, and the contamination of results by additional treatment outside the intervention provided by some participating pharmacists. |
| **Cullum et al. 2007** | **Low risk**  Concealed, random allocation. No significant baseline differences. | **NA** | **Low risk**  Participants could not be blinded to allocation, however there is no evidence that this knowledge led to any deviations from the intended intervention. | **Some concerns**  Significant attrition rate, with appropriate analysis conducted to estimate effect. | **Some concerns**  Data not available for all participants. It is possible, but considered unlikely, that this missingness could have depended on the true value. | **Low risk**  Outcome assessors were blinded to the treatment allocation of participants | **Low risk**  Results were analysed in accordance with a pre-specified data analysis plan. | **Some concerns**  Some concerns for domains 2.2 and 3 due to the lack of available data for all participants. The study results appear to be reported in a per-protocol fashion, rather than the stated intention to treat the method. |
| **Dwight-Johnson et al. 2011** | **Some concerns**  No information on if allocation was concealed, randomisation occurred via telephone using stratified permuted-block randomization,  **with strata defined by gender and referral source, nil baseline imbalances** | **NA** | **Low risk**  No information on if participants and personnel were not aware of intervention but referrers were aware of intervention. Nil deviations appear to have arisen regardless = low risk  **Appropriate analysis to estimate effect of assignment = low risk** | **Some concerns**  Assignment may not have been appropriately analysed but this doesn't have a substantial impact on the study | **Low risk**  Outcome data is complete. | **Low risk**  Method and measurement of the outcome was appropriate and the same between groups. Outcome assessors were blinded and therefore not aware of the intervention received. | **Low risk**  Result outcomes and analyses were appropriate and the trial was analysed in accordance with a pre-specified plan. | **Some concerns**  Some concerns for domains 1 and part of 2 but overall this doesn’t seem to weigh heavily on the study and its risk of bias as a whole. |
| **Hilty et al. 2007** | **Low risk**  Allocation random via a random number table  No baseline imbalances | **NA** | **Some concerns**  No information about blinding and no side effects experienced due to intervention specifically. No information on if carers/people delivering intervention knew what the participants were assigned to. No information on if there were deviations that affected the outcome. | **Some concerns**  Analysis to estimate the effect of assignment was probably not in full but there doesn't appear to be a substantial impact of this. | **Some concerns**  There was a high dropout rate for this study which may be attributed to health conditions of the participants. There isn't strong evidence that results weren't biassed. It doesn't seem likely that missingness depended on true value. | **Some concern**  Outcome assessors were probably aware of the intervention received although it isn’t clearly stated anywhere. Assessors could have been influenced by this knowledge but it doesn't seem likely. | **High risk**  There have been multiple outcomes measurements and multiple analyses of this data. | **High risk**  Overall high risk of bias as domain 5 is high risk. |
| **Karasz et al. 2021** | **Low risk**  Participants randomised through a computer-generated list of random numbers. Nil baseline imbalances. | **NA** | **Some concerns**  No information on participants or personnel being blinded to the intervention. No information on if there were deviations because of the trial context. | **Some concerns**  No information on appropriate analysis to estimate the effect of assignment but doesn't appear to substantially impact analysis overall. | **Low risk**  Outcome data was readily available for all participants. | **Some concerns**  No information to suggest that outcome assessors weren’t aware of interventions. Assessment therefore could have been influenced by this knowledge but it seems unlikely. | **Some concerns**  Results were appropriate and all included but no information on if the trial analysed in accordance with a pre-specified plan. | **Some concerns**  The study is judged to raise some concerns in at least one domain for this result, but not to be at high risk of bias for any  domain**.** |
| **Kay-Lambkin et al. 2012** | **Some concerns**  No information on how participants were randomised. No information on if the allocation sequence was concealed or not. No indications in baseline data that there were problematic imbalances. | **NA** | **Low risk**  No information on if participants were blinded but personnel were blinded. Probably no to both therefore. | **High risk**  There was not an appropriate analysis to estimate the effect of the assignment and this appears to have substantially impacted this study. | **Low risk**  163/274 participants did the 3 month follow up - only these participants’ data were reported. | **Low risk**  The method of measuring outcome was appropriate and did not differ between groups. The outcome assessors were not aware of intervention received as they were blinded. | **Some concerns**  Multiple analyses of the data. | **High risk**  High risk overall as D2 had a high risk component. |
| **Pradeep et al. 2014** | **Some concerns**  No information on exactly how randomisation was undertaken, no obvious imbalances in baseline data. | **Low risk**  Randomisation occurred prior to recruitment. However, recruitment was blinded to allocation and unlikely to have been affected. Nil significant baseline imbalances to suggest differential recruitment. | **Low risk**  No information on if participants were aware of intervention, personnel were blinded. No obvious deviations. Appropriate analysis. | **Low risk**  Control group used but no mention specifically of if participants were blinded. Non-protocol interventions were balances and nil failure or non-adherence that affected the outcome. | **Some concerns**  Not all participants were present for follow up and there is no evidence that the results have not been biased as only the participants at follow up were used at baseline. It seems unlikely that missingness depended on true value - some concerns. | **Low risk**  Person conducting the studies was blinded to which villages related to which treatment option. The trained physician involved was also blinded. | **Some concerns**  No information on if the results were analysed by blinded researchers. There is potential for multiple analyses of the data. | **Some concerns**  Three domains with some concerns as their rating. Nil high concerns therefore some concerns overall rating. |
| **Ransom et al. 2008** | **Some concerns**  No information on how randomisation occurred. | **NA** | **High risk**  No information on participants or personnel being blinded to the intervention. It appears that deviations affecting the outcome arose and were not balanced between groups. | **Some concerns**  There was an inappropriate analysis to estimate the effect of assignment but this did not have a major impact on the study results. | **High risk**  There was substantial missing outcome data with no evidence that this was not biassed. It seems likely that missingness depended on true value. | **High risk**  No information on who was blinded in this study – participants filled out their own pre and post intervention data and mailed it to the researchers. Method of measurement was appropriate. Unknown if assessment could have been influenced by knowledge of intervention received. Measurement between groups could have differed due to participants filing out their own data as opposed to a trained professional collecting information. | **Some concerns**  No indication that the data collected was analysed in different ways or that there were different numerical results being used. No indication of the contrary either. | **High risk**  Multiple high risk domains indicates that this study has an overall high risk of bias. |
| **Robinson-Whelen et al. 2007** | **Some concerns**  SPSS was used to randomise participants. No information on if this was concealed but baseline data does not suggest this was a problem. | **NA** | **Some concerns**  No information on if personnel were aware of intervention. No information on deviations arising because of trial context. | **High risk**  Minimal information to confirm the details required for this domain. | **Some concerns**  Not all participants had outcome data reported. There was no evidence that these results weren’t biassed but it seems unlikely that missingness depended on true value. | **Some concerns**  Measurement and method of measurement were appropriate and the same between groups. No information on if assessors were aware of the intervention received and it is likely that this could have impacted results. | **High risk**  Varying analyses were undertaken, and the original analyses plans were changed due to unforeseen circumstances. | **High risk**  Multiple high risk domains indicates that this study has an overall high risk of bias. |
| **Sangraula et al. 2020** | **Low risk**  Names of the two Village Development Committees (VDCs) were written on cards and placed into a hat. The District Public Health Officer (DPHO) was to draw one card from the hat which would be allocated as the intervention arm and the remaining card would be allocated to the control arm. No concerning baseline imbalances. | **Low risk**  Randomisation occurred prior to recruitment. However, recruitment was blinded to allocation and unlikely to have been affected. Nil significant baseline imbalances to suggest differential recruitment. | **Some concerns**  Participants are aware of the intervention due to the nature of problem management therapy. The study did not provide information about deviations arising because of the trial context. | **High risk**  Both control and intervention groups were offered ‘family meetings’. There is no information and difficulties in implementing this care. There is also no information about intention to treat analysis or last observation carried forward, likely due to the nature of this study as a pilot study. | **Some concerns**  Only three patients were lost to follow up but there is limited information on the analysis of their data. Since they were lost to follow up, there is no reason for withdrawal. However since the attrition rate is so high it is unlikely these three participants skewed the results. | **Low risk**  The Patient Health Questionnaire (PHQ-9) was used to measure outcome and this is a validated tool. This was used for both groups. The supplementary information provides detailed information about blinding including that the statistician was blinded to the intervention status of the patients. | **Some concerns**  Only one set of results is shown but no information on whether multiple data analyses were performed. | **High risk**  High risk domain indicates that this study has an overall high risk of bias. |
| **Scogin et al. 2018** | **Some concerns**  Study stated that participants were randomised but there is no information on randomisation method eg computer generated randomisation. The study states that baseline characteristics between intervention and control groups were compared to establish that randomisation was successful so no apparent baseline imbalance. | **NA** | **Some concerns**  Participants, family members and providers are aware of the intervention due to the nature of CBT. The study did not provide information about deviations arising because of the trial context. | **High risk**  Participants, family members and providers are aware of the intervention due to the nature of CBT. Both intervention and control groups received normal physician care for depression and insomnia but there is no information about any failures in implementation that could have impacted the outcome. Additionally, there is no information on data analysis and whether intention to treat analysis was used. | **Some concerns**  Some patients withdrew or were lost to follow up. There is limited information on how their data was incorporated into the analysis. | **Low risk**  Study used the HAM-D score which is a validated scale for depression. This was used for both groups. Study states that the project research assistants were blind to the participants' treatment conditions and that they conducted all assessments. | **Some concerns**  Multiple analyses of data were performed. | **High risk**  One high risk domain and multiple domains of some concern lead to an overall high risk rating. |
| **Xie et al. 2019** | **Low risk**  Patients were randomly numbered using a random number table and then divided into two groups. The baseline characteristics show no major imbalances. | **NA** | **Some concerns**  Participants are aware of the intervention due to the nature of CBT. The study did not provide information about deviations arising because of the trial context. | **High risk**  Regular care was standardised by investigators to ensure both groups received similar usual care. Although the attrition rate is high, there is no information about failures in implementing treatment that could have impacted the outcome. There is also no information on whether intention to treat analysis was used. | **Some concerns**  A total of 7 patients were lost. Reasons for withdrawal were documented and it is possible but unlikely this was related to depression. Only participants with complete data were included in the analysis. | **Some concerns**  The Geriatric Depression Scale (GDS) was used to measure outcome and this is a validated tool. This was used for both intervention and control groups. There is limited information about blinding of assessors and it is possible that they could have been influenced by knowledge of intervention. | **Some concerns**  Only one data set is shown but there is no information about whether this data was selected from multiple data analyses. | **High risk**  One high risk rating and multiple concerns rating leads to this being an overall high ROB. |
| **Yuan et al. 2020** | **Low risk**  Random block sizes used for randomisation. Participants blinded to the treatment arm until after allocation. | **NA** | **Low risk**  Only people blinded were assessors and data analysts. Participants could not be blinded to intervention, however there is no evidence this affected results.Analysed using intention to treat. | **Some concerns**  Participants and implementers were aware of assigned intervention. There was good adherence across both groups. Intention to treat analysis used. There was no assessment of homogeneity or validity of the lay-counsellors interventions. | **Low risk**  No information on retention rates, however intention to treat analysis was used. | **Low risk**  Multiple outcome measures (parallel screening) used to lower false positive rates. Assessors were blind to participants’ allocation. | **Some concerns**  All eligible analyses of the data included, however, no locatable study protocol or analysis plan. | **Some concerns**  Concerns raised in D2.2 and 5. |

##

## **Supplementary Table 3 - Definitions of rurality**

| **Country of Origin** | **Administrative definition of rurality** | **Articles relevant** |
| --- | --- | --- |
| Australia | Various, however the ASGS classifies rurality by population size and density  A geographic-based definition has been used by the Australian Parliament and the Regional Australia Institute (1)   - All the towns, cities, and areas outside Australia’s largest capital cities; Sydney, Melbourne, Brisbane, Adelaide, Perth and Canberra   The Australian Bureau of Statistics uses a population-based   - The ABS Section of State (SOS) Structure of the ASGS defines Major Urban and Minor Urban areas as those with a population >100,000 and between 1000-100,000 people respectively (2) - HOWEVER, this seems to just be how the ABS defines a township and is less applicable for use than the geographic-based definition | Almeida 2020 |
|  |  | Kay-Lambkin 2012 |
|  |  | Crockett 2006 |
| USA | Primarily a based on population size and density, urban/nonurban land use, and type of dwelling   - Urbanised Areas are defined as those containing 50,000 or more people, with any region outside of these areas being classed as “rural”. (3) | Scogin 2018 |
|  |  | Dwight-Johnson 2011 |
|  |  | Ransom 2008 |
|  |  | Hilty 2007 |
|  |  | Robinson-Whelen 2007 |
| Bangladesh | Difficult to identify, but appears to be a population-density based definition (4) | Karasz 2021 |
| Uganda | Population-based   - Urban areas are gazetted cities, municipalities and towns with a population over 2,000 persons - Rural areas are regions with a population of <2000 persons (5) | Bass 2006 |
| India | Population size and density, ratio of agricultural/non-agricultural pursuits   - Urban is defined by the census as all places that have:   - Population ≥5,000   - ≥75% of the male main working population engaged in non-agricultural work   - Population density ≥400 per square kilometre - Other areas outside of statutory limits for a city were included within the urban definition if they were contiguous with a statutory town and had infrastructure such as electricity, education institutions and banks - All other non-urban areas were classified as rural (5) | Pradeep 2014 |
| Iraq | Difficult to identify, but appears to be based on population-density and agricultural land usage (6) | Bolton 2014 |
| China | Primarily defined by population-density, infrastructure usage and amenities   - There is very little current information on this that has been published in English - The 2000 census defined urban as districts with an average population density of ≥1,500 persons per square kilometre, other population in suburban-district units and township-level units meeting criteria such as "contiguous built-up area," or being the location of the local government - Those living in villages or towns in outer urban and suburban areas that are directly connected to municipal infrastructure, and that receive public services from urban municipalities, were also included as urban residents. (5) | Chen 2022 |
|  |  | Yuan 2020 |
|  |  | Xie 2019 |
| United Kingdom | Appears to be a population-based definition   - England and Wales: rural areas are made up of villages, hamlets and isolated dwellings - Scotland: same definition as for England and Wales, with accessible or very remote small towns also included. - Non-rural is defined as urban areas with a population of ≥10,000, and town fringe areas. Scotland classifies both large and small urban areas as non-rural. (5) | Cullum 2007 |
| Nepal | Primarily an arbitrary administrative definition (ie 4 administrative regions out of 58 are classed as metropolitan or sub-metropolitan, with all others being rural) (5) | Sangraula 2020 |

**References – Supplementary Table 3**

1. Drum, D. Regions at the ready: Investing in Australia’s future [Internet]. Canberra, ACT: Commonwealth of Australia; 2018 June [cited 2023 November 6]. Available from: https://www.aph.gov.au/Parliamentary_Business/Committees/House/Former_Committees/Regional_Development_and_Decentralisation/RDD/Final_Report/section?id=committees%2Freportrep%2F024136%2F25543
2. Australian Bureau of Statistics. Frequently asked questions: How does the ABS define urban and rural [Internet]. Canberra, ACT: Commonwealth of Australia; 2023 [cited 2023 November 6]. Available from: https://www.abs.gov.au/statistics/statistical-geography/frequently-asked-questions#how-does-the-abs-define-urban-and-rural-
3. Health Resources and Services Administration [Internet]. Rockville, MD: U.S. Department of Health and Human Services; 2022. Defining rural population. 2022 March [cited 2023 November 6]. Available from: https://www.hrsa.gov/rural-health/about-us/what-is-rural#:~:text=How%20does%20the%20Census%20define,of%2050%2C000%20or%20more%20people
4. Trading Economics [Internet]. 2023. Bangladesh: Rural population [Internet]. 2023 [cited 2023 November 6]. Available from: https://tradingeconomics.com/bangladesh/rural-population-percent-of-total-population-wb-data.html
5. International Labour Organization [Internet]. Geneva: ILO; No date. Inventory of official national-level statistical definitions for rural/urban areas. No date [cited 2023 November 6]. Available from: https://www.ilo.org/wcmsp5/groups/public/---dgreports/---stat/documents/genericdocument/wcms_389373.pdf
6. Phillips DG. Rural-to-urban migration in Iraq. Economic Development and Cultural Change. 1959;7(4):405–21. doi:10.1086/449813
